# Current Trends in Gluten-Free Biscuit Formulation Using Rice Flour Enriched with Chestnut Flour and Fruit Powders

**DOI:** 10.3390/foods14234074

**Published:** 2025-11-27

**Authors:** Daniela Stoin, Mariana-Atena Poiana, Ersilia Alexa, Ileana Cocan, Monica Negrea, Calin Jianu, Isidora Radulov, Mariana Suba, Catalin Ianasi

**Affiliations:** 1 Faculty of Food Engineering, University of Life Sciences “King Mihai I” from Timisoara, Aradului Street No. 119, 300645 Timisoara, Romania; danielastoin@usvt.ro (D.S.); ersiliaalexa@usvt.ro (E.A.); ileanacocan@usvt.ro (I.C.); monicanegrea@usvt.ro (M.N.); calinjianu@usvt.ro (C.J.); 2 Food Science Research Center, University of Life Sciences “King Mihai I” from Timisoara, Aradului Street No. 119, 300645 Timisoara, Romania; 3 Faculty of Agriculture, University of Life Sciences “King Mihai I” from Timisoara, Aradului Street No. 119, 300645 Timisoara, Romania; isidora_radulov@usvt.ro; 4 Romanian Academy, “Coriolan Dragulescu” Institute of Chemistry, Mihai Viteazu No. 24, 300223 Timisoara, Romania; marianasuba@gmail.com (M.S.); ianasic@acad-icht.tm.edu.ro (C.I.)

**Keywords:** rice flour, chestnut flour, fruit powders, gluten-free biscuits, physico-chemical and structural properties, functional profile

## Abstract

In response to the increasing consumer demand for healthier diets and the needs of individuals with gluten intolerance, chestnut flour (CF) emerges as a valuable unconventional ingredient for sustainable and functional nutrition. This study evaluated the nutritional, phytochemical, and functional properties of gluten-free biscuits formulated with whole rice flour (RF), CF, and their mixtures, where RF was replaced by CF at 0% (control), 10%, 30%, 70%, 90%, and 100% (*w*/*w*). In addition, in the 50% CF formulation, 5% of RF was substituted with fruit powders rich in phenolic compounds and recognized as fortifying agents, such as chokeberry (CP), açaí (AP), and blueberry (BP). Proximate composition, macro- and microelement content, total phenolic content (TPC), total flavonoid content (TFC), and antioxidant activity (DPPH and FRAP assays) were determined for the individual flours, composite flours, fruit powders, and biscuit formulations. Structural characteristics were assessed using Small- and Wide-Angle X-ray Scattering (SAXS/WAXS) analysis and Fourier Transform Infrared Spectroscopy (FTIR). Results showed that CF incorporation enhanced both the nutritional and functional profile of flours and biscuits, increasing protein, fiber, lipid, and mineral contents while reducing carbohydrates, and improving TPC, TFC, DPPH, and FRAP values. Fortification with 5% CP, AP, or BP further boosted the phytochemical content of the biscuits, with the chokeberry-enriched sample exhibiting the highest TPC (348.88 mg GAE/100 g d.s.), TFC (253.82 mg QE/100 g d.s.), DPPH (50.36%), and FRAP (21.07 μM Fe^2+^/g d.s.). The combination of 50% CF and 5% CP provided dual benefits, significant bioactive enrichment alongside the preservation of desirable technological properties. Complementary SAXS/WAXS and FTIR analyses indicated that CF and fruit powders enhanced molecular interactions and matrix cohesion, which may contribute to improved texture and antioxidant potential of the biscuits. Overall, this formulation offers a promising and practical approach to developing functional gluten-free biscuits with enhanced nutritional, phytochemical, functional, and structural characteristics.

## 1. Introduction

Today, global food systems face unprecedented challenges, including climate change, population growth, urbanization, resource scarcity, and malnutrition. These factors, along with the poor nutritional quality of many diets, exacerbate public health concerns. In this context, research in food technology and nutrition increasingly focuses on identifying and valorizing unconventional raw materials to diversify the food supply and develop functional ingredients that support human health. A central area of investigation concerns the development of innovative products for people with food intolerances, particularly those with gluten intolerance or celiac disease [1]. Current studies aim to enhance both the nutritional and sensory profile of gluten-free foods while addressing technological challenges related to processing and product quality. Celiac disease, an autoimmune disorder induced by gluten, can only be managed through a lifelong gluten-free diet [2].

Rice flour is widely used as a base for gluten-free products due to its absence of gluten proteins, hypoallergenic properties, and good digestibility [3]. However, it is low in protein, lipids, fiber, iron, B vitamins, and folic acid, while being relatively high in carbohydrates [4]. To address these nutritional shortcomings, it is often combined with other gluten-free flours or functional ingredients to improve both nutritional quality and technological performance [5,6]. Chestnut flour represents a promising functional ingredient and has been explored in a few previous studies for the formulation of gluten-free cookies [5,6,7] due to its unique nutritional and phytochemical profile. It contains high-quality proteins with essential amino acids (4–7 g/100 g), resistant starch (50–60 g/100 g), dietary fiber (4–10 g/100 g), and a low lipid content (2–4 g/100 g, predominantly unsaturated) [5,8]. Additionally, chestnut flour is a valuable source of vitamins (B, C, and E); minerals such as potassium, phosphorus, and magnesium; as well as phenolic compounds, pholates, gallic, and ellagic acid [9]. Its proteins are rich in essential amino acids including aspartic acid, asparagine, and glutamic acid [10]. These attributes underline the potential of chestnut flour to replace conventional cereal and tuber starches, thereby contributing to technological improvements in food products such as enhanced texture, rheology, gelation, and moisture retention [11]. Moreover, the high fiber and sugar contents of chestnut flour offer additional functionality: fibers contribute to emulsification, stabilization, and thickening of dough, while natural sugars improve color and flavor during baking. Nevertheless, the use of chestnut flour in isolation often leads to technological drawbacks such as low product volume, dark coloration, and off-flavours. To mitigate these issues, blending chestnut flour with rice flour has been widely recommended as a strategy to enhance product quality [12,13]. As highlighted by studies conducted by Torra et al. [6] and Silav-Tuzlu and Tacer-Caba [14], the incorporation of chestnut flour into gluten-free biscuit formulations significantly enhances the rheological and sensory properties of the final product. Furthermore, the resulting biscuits exhibited high total phenolic content and antioxidant activity, indicating superior oxidative stability.

Extensive research has demonstrated the versatility of chestnut flour in bakery applications, demonstrating its successful incorporation into a wide range of flour products, including cookies [5,15], biscuits [16], wafers [17], bread [10], muffins [18], and pasta [19] in proportions of up to 60% of the flour blend. Consequently, the utilisation of chestnut flour emerges as a viable approach for the production of gluten-free bakery products that possess high functional value. Beyond its technological utility, chestnut flour provides significant health benefits. Its vitamin and mineral content contributes to immune system reinforcement, prevention of osteoporosis, cardiovascular protection, and reduction of oxidative stress-related damage. The antioxidant compounds present in chestnuts are capable of neutralizing free radicals and preventing oxidative damage to biomolecules such as proteins, DNA, and lipids, thereby potentially reducing the risk of chronic degenerative diseases [6].

In addition to flour-based alternatives, the incorporation of fruit powders represents an effective strategy to enhance the nutritional and functional properties of biscuit formulations, aligning with consumer demand for health-oriented and functional foods [20]. Powders derived from chokeberry *(Aronia melanocarpa*) [21], açaí (*Euterpe oleracea*) [22], and blueberry (*Vaccinium myrtillus* L.) [23] are rich in vitamins, minerals, dietary fiber, and polyphenolic compounds. Chokeberry provides vitamins C, K, E, as well as B-group vitamins; minerals such as calcium (Ca), zinc (Zn), iron (Fe), magnesium (Mg), and potassium (K); insoluble dietary fiber including lignin, cellulose, hemicellulose; and polyphenols such as anthocyanins, proanthocyanidins, phenolic acids, and flavonols [24]. Consumption of chokeberry has been associated with immune modulation, cardiovascular and ocular health obesity, inflammation, thrombosis, and antibacterial effects [25]. Açaí contains vitamins C, E, A, K, and B-complex; minerals (Ca, Fe, K, Mg, Zn, P); carotenoids; flavonoids; tocopherols; and dietary fiber, supporting antioxidant defense, immune function, cardiovascular protection, and metabolic regulation [26]. Blueberries provide dietary fiber; vitamins C, A, and K; minerals (K, Se, Ca, P, Zn, Mg, Mn, Fe); and flavonoid compounds, contributing to the prevention of cancer, cardiovascular disorders, diabetes, and neurodegenerative diseases [27,28]. These fruit powders enhance the nutritional and functional profile of biscuits by providing fiber, polyphenols, minerals, and antioxidant activity, while also positively influencing sensory attributes such as color, texture, and aroma, without compromising overall product acceptability [21,22,23,29].

The technical justification for our study is based on previous research demonstrating that average substitution rates of chestnut flour (CF) (20–60%) lead to significant improvement in rheological and sensory properties [5], as well as an increase in protein and ash content, while maintaining the sensory characteristics of the biscuits [30]. In contrast, higher substitution levels (>60%) negatively affected dough workability, leading to reduced technological performance and poorer sensory attributes [5,30]. Similarly, the incorporation of fruit powders such as chokeberry [23,31], açaí [22], and blueberry [29] in a proportion of 5–10% led to an improvement in the phytochemical profile, sensory characteristics, and consumer acceptance of the biscuits. At the same time, higher levels of fruit powder inclusion (>15%) resulted in a reduction in biscuit quality, attributed to increased acidity, bitter taste, and darker color [22,29,31]. Although the positive impact of chestnut flour on enhancing the nutritional quality of rice-based gluten-free products has been demonstrated, no comprehensive studies have investigated how replacing rice flour with varying amounts of chestnut flour affects the nutritional and phytochemical characteristics of the final products, nor the combined effect of incorporating chestnut flour together with fruit powders.

In this context, the aim of this study was to explore the nutritional and phytochemical potential of rice flour, chestnut flour, and their composite mixtures as unconventional raw materials in gluten-free biscuit formulation. The experimental biscuit formulations, developed with varying proportions of rice flour (RF) and chestnut flour (CF), were evaluated for their nutritional, phytochemical, physical, and structural characteristics to determine their suitability as components of a sustainable and health-oriented diet. The rice flour (RF)–chestnut flour (CF) combination (50:50) was further enriched with chokeberry, açaí, and blueberry powders to evaluate their potential to enhance the nutritional and functional quality of the biscuits. This approach fills a gap in the literature concerning the combined use of chestnut flour and bioactive fruit powders, with the goal of enhancing the nutritional value, functional properties, and technological performance of the products. The practical objective of the study was to provide guidance for the development of nutritionally enriched gluten-free biscuits with enhanced bioactive content, antioxidant capacity, and overall product quality.

## 2. Materials and Methods

### 2.1. Biscuit Ingredients and Manufacturing Process

CF produced by Paleolit (Budapest, Hungary) was purchased from Cocomag shop Oradea, Romania, and whole rice flour (RF) was purchased from Solaris shop Timisoara, Romania. Chokeberry (CP), açaí (AP), and blueberry (BP) powders were purchased from Minunea Naturii, a natural products store in Timișoara, Romania.

Based on these flours and powders, two sets of composite flours were prepared. Starting from rice flour (RF) as the base flour, the first set was obtained by partially substituting it with chestnut flour (CF) at levels of 0% (control), 10%, 30%, 50%, 70%, 90%, and 100%, labeled as RF, 90RF10CF, 70RF30CF, 50RF50CF, 30RF70CF, 10RF90CF, and CF.

The second set of composite flours was prepared by further substituting RF in the 50RF50CF formulation with 5% fruit powders, namely, chokeberry powder (CP), açaí powder (AP), and blueberry powder (BP), resulting in four additional mixtures: 50RF50CF (control), 45RF50CF5CP, 45RF50CF5AP, and 45RF50CF5BP.

From these two sets of composite flours, gluten-free biscuit formulations were prepared, coded as follows: BRF (control), B90RF10CF, B70RF30CF, B50RF50CF, B30RF70CF, B10RF90CF, and BCF for Set I and B50RF50CF (control), B45RF50CF5CP, B45RF50CF5AP, and B45RF50CF5BP for Set II. The other ingredients from the biscuit manufacturing recipe were acquired from retail stores and consisted of natural sweetener from Stevia (Sly Nutrition, Sweet&Safe, Buzau, Romania), butter with 82% fat (Napolact, Cluj-Napoca, Romania), baking powder (Dr. Oetker, Arges, Romania) and eggs (Auchan supermarket, Timisoara, Romania).

**Figure 1:**
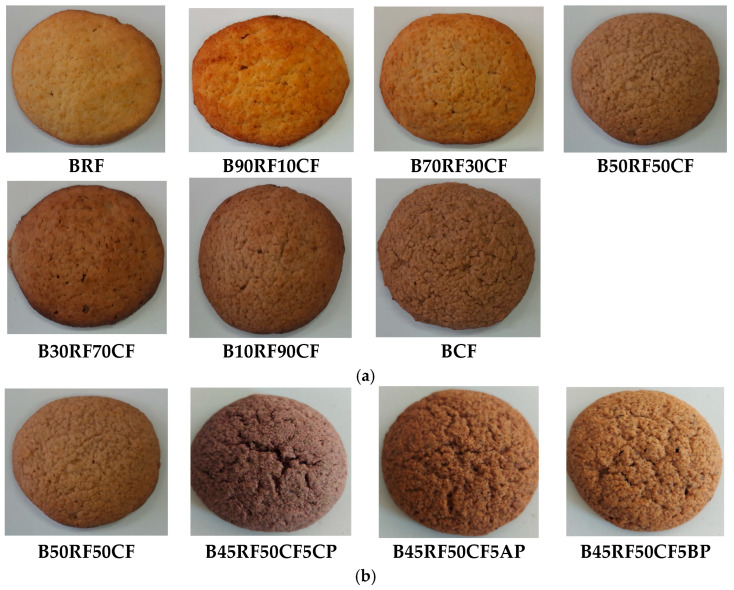
Biscuit formulations: (**a**) from rice flour (RF) with chestnut flour (CF) and (**b**) from rice flour with chestnut flour plus fruit powders. Set I (BRF, B90RF10CF, B70RF30CF, B50RF50CF, B30RF70CF, B10RF90CF, BCF): biscuits from rice flour with 0%, 10%, 30%, 50%, 70%, 90%, and 100% chestnut flour. Set II (B50RF50CF, B45RF50CF5CP, B45RF50CF5AP, B45RF50CF5BP): biscuits from rice flour with 50% CF plus 5% fruit powders—chokeberry powder (CP), açaí powder (AP) and blueberry powder (BP).

The gluten-free biscuit formulas were prepared following the technological process described by Torra et al. [6] with minor modifications. Table 1 shows the recipe of the gluten-free biscuit formulations and the specific parameters of the manufacturing process. To prepare the dough, butter and sweetener were mixed for 3 min with a Maxima Planetary Mixer MPM 7 (Nijverheidsweg, Rp Mijdrecht, The Netherlands); then, the eggs were added and it was mixed again for 3 min at speed 3. Then, the flour, baking powder, and salt were added and mixed at speed 3 for 3 min until a malleable and homogeneous dough was formed, which was covered with polyethylene film and left at 4 °C for 60 min. The resulting dough was rolled to a thickness of 6 mm and cut with a circular cookie shaper with a 6 cm diameter cutter. After baking at 170 °C for 20 min in a professional convection oven (FM RXB 610 V7; 13,650 W, 400 V, 50/60 Hz; FM Industrial S.A., Lucena, Córdoba, Spain), the biscuits were cooled at room temperature for 12 h. From each batch of biscuits, a representative sample was randomly collected, sealed in polypropylene bags, and stored at −18 °C until chemical analyses were conducted. The remaining samples were transferred to food-grade plastic containers and stored for further analysis. For each formulation, three independent replicates (*n* = 3) were prepared, and three samples were obtained from each replicate. Figure 1 illustrates the biscuit samples.

### 2.2. Proximate Composition of Composite Flours, Fruit Powders, and Biscuits

The proximate composition of composite flours, fruit powders, and biscuit formulations was determined using AOAC standard methods [32] for moisture (Official Method 925.10), lipid (Official Method 922.06), protein (Official Method 920.87), and crude fiber (Official Method 991.43) content while ISO Method 2171:2023 [33] was used for ash content. The total carbohydrate content of samples was determined based on the relationship shown in equation 1, by calculating the percentage remaining after removing all other measured components such as lipids, protein, ash and moisture, like Borșa et al. [34].

Total carbohydrates (%) = 100 − [lipids (%) + proteins (%) + ash (%) + moisture (%)](1)

The energy value of the samples analyzed was calculated using Equation (2), in accordance with Silav-Tuzlu et al. [14], by summing the calorie intake of each nutrient (carbohydrate, protein and lipid), taking into account that 1 g of lipid = 9 kcal, 1 g protein = 4 kcal and 1 g carbohydrate = 4 kcal. All samples were measured in triplicate.

(2) Energy value (kcal/100 g)=lipids (%)×9+proteins (%)×4+carbohydrates (%)×4

### 2.3. Physical Properties of Biscuit Formulations

The physical properties of the biscuit samples were determined according to the methods described by Drakos et al. [35]. The biscuits were selected randomly for physical analyses (weight, diameter, thickness, and spread ratio). Using an electronic balance (Mettler, Gießen, Germany), the weight of three biscuits from each sample was determined and the average weight was expressed in grams (g). The diameter of six biscuits was measured using a ruler and expressed in millimeters (mm). The diameter of each biscuit was measured at 90°, 180°, 270°, and 360°, and the mean value was recorded. The thickness was determined by placing three biscuits on top of each other, then placing them in a different order and measuring them to obtain the average in millimeters (mm) and recording the average of the measurements. The spread ratio of the biscuits was determined by dividing the average value of the diameter by the average value of the thickness of the same biscuits. All analyses were performed in triplicate.

### 2.4. Macro- and Microelement Content of Composite Flours, Fruit Powders, and Biscuits

The evaluation of the macro- (K, P, Mg and Ca) and microelement (Fe, Zn, Mn and Cu) content of the analyzed samples was performed by atomic absorption spectroscopy (AAS) using a Varian 220 FAA spectrometer (Palo Alto, CA, USA) after calcination of the samples, according to the methods described by Plustea et al. [36]. Specifically, 3 g of each sample were calcined at 650 °C in a muffle furnace (Nabertherm GmbH, Lilienthal, Germany). The resulting ash was dissolved in 20% HCl and diluted to a final volume of 20 mL in a volumetric flask, and the solution was used to determine macro- and microelements. A mixed standard solution (ICP Multi Element Standard Solution IV CertiPUR) was used to perform the calibration curve. All chemicals and solvents were of analytical grade. Results were expressed in ppm and measurements were performed in triplicate.

### 2.5. Phytochemical Profile of Composite Flours, Fruit Powders, and Biscuits

#### 2.5.1. Preparation of Alcoholic Extracts from Samples

Ethanolic extracts were prepared in order to evaluate the content of total phenolic compounds, total flavonoids and antioxidant activity, using a method adapted from that of Litwinek et al. [37]. Approximately 1.0 g of the sample was mixed with 10 mL of 70% ethanol (*v*/*v*) (SC Chimirec SRL, Bucharest, Romania) and stirred for two hours at room temperature using an IDL magnetic stirrer (IDL GmbH & Co., KG, Nidderau, Germany). After centrifugation at 10,000 rpm for 10 min (rotor radius: 9.5 cm) using a Hettich EBA 21 centrifuge (Andreas Hettich GmbH & Co., KG, Tuttlingen, Germany), the residue was subjected to a second extraction with 70% ethanol for 1 h under continuous stirring. This was followed by a new centrifugation under the same conditions. The resulting two extracts were combined and stored at −20 °C in the dark until analysis. All samples were processed in triplicate, with each replicate analysed independently in subsequent tests.

#### 2.5.2. Assessment of Total Phenolic Content

The total phenolic content (TPC) of the samples was determined using the Folin–Ciocalteu reagent according to the method described by Blanch et al. [38] with some modifications. Prior to analysis, the alcoholic extracts were diluted with distilled water at the following ratios: 1:5 (*v*/*v*) for RF and CF; 1:100 (*v*/*v*) for CP, AP, and BP; 1:10 (*v*/*v*) for composite flours; and 1:5 (*v*/*v*) for biscuit formulations. The Folin–Ciocalteu reagent (2.5 mL; Sigma-Aldrich Chemie GmbH, München, Germany) was added to each filtered extract (0.5 mL), previously diluted 1:10 (*v*/*v*) with distilled water. The samples were treated with 2 mL of 7.5% Na_2_CO_3_ after being maintained at room temperature for 5 min. After incubation for 30 min at 50 °C in an INB500 thermostat (Memmert GmbH, Schwabach, Germany), absorbance was determined at 750 nm using a UV–VIS spectrophotometer (Specord 205; Analytik Jena AG, Jena, Germany), with a blank prepared under the same conditions as the samples. The results were expressed as mg gallic acid equivalents (GAEs) per 100 g of sample. All determinations were performed in triplicate. Standard gallic acid solutions (Fluka, Madrid, Spain) with concentrations ranging from 10 to 100 mg GAE/L were used to generate the calibration curve. Total phenolic content (TPC) was expressed as mg GAE/g.

#### 2.5.3. Assessment of Total Flavonoid Content

Total flavonoid content (TFC) of the samples was estimated using a modified method of Al-Farsi et al. [39]. For each sample, 3 mL of extract was mixed with 4.5 mL of distilled water and 1.0 mL of 0.3% NaNO_2_ solution. After incubation for 6 min at 20 °C, 1 mL of 10% Al(NO_3_)_3_ solution was added. Following an additional 6 min incubation, 10 mL of 4% NaOH (*w*/*w*) solution was added, and the final volume was adjusted to 25 mL with 60% ethanol (*v*/*v*). After 15 min, absorbance was measured at 510 nm against a control containing 70% (*v*/*v*) ethanol using a UV–VIS spectrophotometer (Specord 205; Analytik Jena AG, Jena, Germany). All measurements were performed in triplicate and results were expressed as mg quercetin equivalents (QEs)/100 g d.s. The calibration curve was prepared using standard quercetin solutions with concentrations ranging from 0.5 to 50 µg/mL.

#### 2.5.4. Assessment of Antioxidant Capacity

The antioxidant activity of the samples was investigated by 1,1-diphenyl-2-picrylhydrazyl (DPPH) radical scavenging assay and ferric ion reducing antioxidant power (FRAP) assay.

##### DPPH (1,1-diphenyl-2-picryl-hydrazyl) Assay

The antioxidant activity (AA) of the samples (rice flour, chestnut flour, fruit powders, composite flours, and their corresponding biscuit formulations) was evaluated using the DPPH assay [40], with a 0.1 mM DPPH solution prepared in 70% (*v*/*v*) ethanol. The previously obtained ethanol extracts were further diluted with 70% (*v*/*v*) ethanol at a ratio of 1:5 (*v*/*v*) for RF, CF, composite flours and biscuit formulations and 1:100 (*v*/*v*) for CP, AP, and BP. Subsequently, 1.0 mL of each diluted extract was mixed with 2.5 mL of 0.1 mM DPPH solution in 70% (*v*/*v*) ethanol. The mixtures were homogenized using a hot plate stirrer (IDL, IDL GmbH & Co KG, Nidderau, Germany) and incubated in the dark for 30 min at 20 °C. Absorbance was measured at 517 nm against 70% (*v*/*v*) ethanol. A control sample, consisting of 1 mL of 70% (*v*/*v*) ethanol mixed with 2.5 mL of 0.1 mM DPPH solution in 70% (*v*/*v*) ethanol, was prepared under the same conditions. Three readings were taken for each sample. The percentage of inhibition of DPPH was calculated using Equation (3):

(3) $$\mathrm{DPPH}\;\mathrm{inhibition}\;(\%)=\frac{A_{\mathrm{control}}-A_{\mathrm{sample}}}{A_{\mathrm{control}}}\times 100$$

where A_control_ represents the absorbance of the control and A_sample_ is the absorbance measured in the presence of the tested sample. All experimental analyses were performed in triplicate.

##### FRAP Assay

The antioxidant capacity of the samples (rice flour, chestnut flour, fruit powders, composite flours, and their corresponding biscuit formulations) was evaluated using the Ferric Reducing Antioxidant Power (FRAP) assay, following the principle established by Pop et al. [41] with slight adaptations. The method relies on the conversion of ferric ions (Fe^3+^) into their ferrous state (Fe^2+^) through the action of electron-donating compounds. In the presence of 2,4,6-tripyridyl-s-triazine (TPTZ), the reduced Fe^2+^ forms a stable blue-colored complex, which displays a strong absorption maximum at 593 nm [42]. The increase in absorbance at this wavelength was monitored using a Specord 200 UV-Vis double-beam spectrophotometer (Analytik Jena Inc., Jena, Germany). The FRAP reagent was freshly prepared by mixing 100 mL of acetate buffer (pH 3.6) with 10 mL of TPTZ solution (10 mM in 40 mM HCl) and 10 mL of FeCl_3_·6H_2_O solution (20 mM). Before measurement, the previously obtained hydroalcoholic extracts of the tested samples were diluted with distilled water as follows: 1:5 (*v*/*v*) for chestnut flour (CF), rice flour (RF), their mixtures, and corresponding biscuit formulations; 1:200 (*v*/*v*) for fruit powders (CP, BP, AP); and 1:10 (*v*/*v*) for composite flours containing 5% fruit powder as well as their corresponding biscuit formulations. For the assay, 0.5 mL of each diluted extract was combined with 2.5 mL of the FRAP reagent and incubated at 37 °C for 30 min. Absorbance was then recorded at 593 nm against a blank prepared under identical conditions. The reducing capacity was expressed as µM Fe^2+^ equivalents per g dry substance (d.s.), using a calibration curve constructed with FeSO_4_·7H_2_O standard solutions (0.05–0.5 µM Fe^2+^ equivalents/mL). All experiments were carried out in triplicate, and the data were reported as mean values ± standard deviation (SD).

### 2.6. Characterization of Flours and Fruit Powders Using Fourier Transform Infrared Spectroscopy (FTIR)

FTIR-ATR spectroscopy (Attenuated Total Reflectance Fourier Transform Infra-red spectroscopy) was applied to RF, CF, fruit powders (CP, AP, BP), and biscuit formulations (BRF, BCF, B45RF50CF5CP, B45RF50CF5AP, B45RF50CF5BP) to identify characteristic functional groups and detect structural changes resulting from the substitution of RF with CF and fruit powders, allowing a comparative assessment of the matrix composition of the samples. Measurements were carried out at room temperature using a Nicolet™ iS50 FTIR spectrometer (Thermo Fisher Scientific, Waltham, MA, USA) equipped with an ATR crystal. Spectra were recorded in the range of 4000–600 cm^−1^ with 32 scans at a resolution of 4 cm^−1^. The analysis commenced with a preliminary heating step, commonly referred to as pre-drying, during which the sample was maintained at 40 °C overnight. This step aimed to remove residual moisture that could potentially interfere with subsequent analyses, particularly the spectroscopic measurements. Following the drying process, the sample was directly positioned on the ATR crystal of the FTIR spectrometer for analysis.

### 2.7. Small- and Wide-Angle X-Ray Scattering (SAXS/WAXS) of Flours and Fruit Powders

SAXS/WAXS analyses were used to evaluate the crystalline organization of starch and protein structures in RF, CF, fruit powders (CP, AP, BP), and biscuit formulations, aiming to reveal structural changes induced by the substitution of RF with CF and fruit powders. Measurements were performed using a Xenocs Xeuss 3.0 system (Xenocs SAS, Grenoble, France) equipped with a Cu Genix 3D source and a Dectris Eiger2 Si 1M detector. Analyses were conducted under vacuum at room temperature with Kapton film. The sample-to-detector distances were set to 1800 mm for SAXS and 45 mm for WAXS, using H-Flux collimation and an acquisition time of 300 s. Data were processed with the XSACT software (version 2.10; Xenocs, Grenoble, France). SAXS fitting was carried out in the Guinier region (0.01–0.1 A^−1^), assuming spherical particles and applying the Guinier equation:

(4) $$I(q)=\exp\left(\frac{-q\times {\mathrm{Rg}}^{2}}{3}\right)$$

where I(q) is the scattering intensity, q represents the scatering vector and Rg is the radius of gyration.

### 2.8. Statistical Analysis

The experimental data were obtained from three independent studies, each performed in triplicate. Results are presented as mean ± standard deviation (SD). Statistical differences between formulations were assessed using one-way analysis of variance (ANOVA), followed by Tukey’s post hoc test for multiple comparisons. The homogeneity of variances was evaluated using Levene’s test. The assumptions of homogeneity, normality (or approximate normality) of residuals, and independence were verified and satisfied. Statistical significance was accepted at *p* < 0.05.

## 3. Results and Discussion

### 3.1. Proximate Composition of Composite Flours, Fruit Powders, and Biscuits

The proximate composition of RF, CF, composite flours, CP, AP, BP, and biscuit formulations is presented in Table 2 and Table 3. RF and CF showed significant differences (*p* < 0.05) in proximate components, which is also reflected in the proximate composition of the 90RF10CF–10RF90CF studied mixtures. The moisture content of CF was lower (7.42%) than that of RF (10.33%). CF, like other unconventional flours, is characterized by a relatively high protein content (6.71%) compared to RF (5.75%), as well as higher levels of lipids (3.79% vs. 1.65%), ash (2.79% vs. 2.57%), and crude fiber (9.23% vs. 2.65%). These compositional differences make CF a promising raw material from a nutritional perspective, providing a valuable source of proteins, lipids, minerals, and dietary fiber for human nutrition. The results obtained in terms of proximal CF composition are close to those reported by Raczyk et al., Demirkesen et al., and Hegazy et al. [10,43,44], who reported moisture values ranging from 4.75 to 10.79%, protein from 4.61% to 6.93%, lipid from 3.78% to 4.39%, ash from 1.98% to 2.44% and dietary fiber from 3.09% to 12.49%. The carbohydrate content of CF was 79.28% compared to 79.70% of RF, a value close to that reported by [10], as well as that reported by [6], who reported carbohydrate content values between 68.02% and 71.10%. The elevated carbohydrate content of CF is primarily attributed to the high starch concentration in chestnuts [10]. The composite flours exhibited intermediate proximate compositions depending on the RF–CF ratio, with protein levels ranging from 5.85% to 6.62%, lipids from 1.86% to 3.58%, minerals from 2.59% to 2.77%, and fibers from 3.31% to 8.57%, in agreement with previous studies [6,10,43,44]. A proportional decrease in moisture and carbohydrate contents was observed with increasing CF levels (from 10.04% and 79.66% in 90RF10CF to 7.71% and 79.32% in 10RF90CF, respectively), suggesting that higher CF incorporation may contribute to an extended shelf life of biscuits.

The study on CP, AP, and BP revealed significant differences (*p* < 0.05) in their proximate composition, also reflected in the analyzed flour mixtures (Table 2). CP showed the lowest moisture content (2.86%) compared to AP (5.56%) and BP (7.90%), indicating greater powder stability and longer shelf life under controlled storage conditions [24]. As presented in Table 2, the three fruit powders contained appreciable amounts of protein (CP: 6.66%; AP: 9.02%; BP: 7.28%), lipids (CP: 4.45%; AP: 4.11%; BP: 4.13%), ash (CP: 2.46%; AP: 4.87%; BP: 4.03%), and fiber (CP: 18.22%; AP: 20.30%; BP: 14.14%).

Carbohydrate levels ranged from 35.44% (AP) to 83.57% (CP), corresponding to energy values between 372.93 and 583.83 kcal/100 g. Overall, these findings highlight the rich nutritional composition of the powders, supporting their potential application as functional ingredients in food product development. The proximate composition of AP was higher than the values reported by Loubet Filho et al. [45], who, for freeze-dried AP, reported lower values for moisture (1.17%), protein (5.95%), lipids (42.87%), and ash (1.23%), but a higher fiber content (27.36%). A similar discrepancy was observed for CP compared with the values reported by Jeong et al. [46], who reported higher moisture (13%) and lower levels of lipids (0.33%), protein (6.51%), ash (0.6%), and fiber (16.15%). In the case of BP, differences in proximate composition were also noted compared with Uribe et al. [47], who reported higher moisture (13.68%) and lower levels of protein (3.70%), lipids (1.62%), ash (1.69%), and fiber (4.19%). These discrepancies may be attributed to several factors, including the type of powder, fruit ripeness, harvesting methods, and processing, storage, and handling conditions [24]. The 45RF50CF5CP, 45RF50CF5AP, and 45RF50CF5BP mixtures, obtained by substituting 5% of the 50RF50CF blend with each powder, exhibited compositional changes depending on the powder type. Protein, lipid, ash, and fiber contents slightly increased in all fortified blends, most notably in 45RF50CF5AP, consistent with the higher nutrient levels in AP [46,47]. These modifications improved the nutritional quality of the mixtures and confirmed their potential as functional food ingredients.

The composite biscuit formulations showed a superior nutritional profile compared to the control (BRF), related to the percentage of RF replaced with CF. Comparative analysis (Table 3) revealed that differences in raw materials significantly affected (*p* < 0.05) the proximate composition of the biscuits. BCF had lower moisture (6.16%) than BRF (8.39%), while samples B90RF10CF–B10RF90CF decreased from 8.16% to 6.39%, slightly higher than the range reported by Hegazy et al. [44] (5.60–6.39%). Protein content increased from 6.96% in B90RF10CF to 7.82% in B10RF90CF, with BCF at 7.93%, higher than BRF (6.88%), consistent with previous reports [6,44]. Lipid content increased from 11.49% (BRF) to 11.87% (B90RF10CF) and 14.93% (B10RF90CF), comparable to values reported for 100% CF products (15.2–17.7%) [6,14]. Ash content ranged from 2.97% to 3.66%, higher in CF-enriched biscuits due to the mineral content of CF (2.79%) and consistent with previous studies [6,14,44]. Crude fiber followed a similar trend, from 3.30% in B90RF10CF to 7.87% in B10RF90CF, with BCF at 8.44%, higher than BRF (2.73%) [6]. Total carbohydrates ranged from 70.04% (B90RF10CF) to 67.20% (B10RF90CF) versus 70.39% in BRF, consistent with literature values (~71%) [6]. Energy values increased with CF content, from 412.36 kcal/100 g in BRF to 434.46 kcal/100 g in B10RF90CF and 436.92 kcal/100 g in BCF, exceeding values reported by Silav-Tuzlu et al. [14] (397.5 kcal/100 g). The effect of CF addition on gluten-free pastry products has been investigated in other studies conducted by Torra et al. [6], Silav-Tuzlu and Tacer-Caba [14], Sharma and Devi [15], and Hegazy et al. [44] in the case of biscuits, Demirkesen [5] in the case of cookies and their results well matched with those reported by us, revealing that as the level of CF substitution increased, all investigated compositional chemical parameters increased. According to the results, the production of bread, cookies, pasta and bakery products seems to be the main applications of chestnut flour. Therefore, CF is an attractive and sustainable ingredient that could be considered for the development of gluten-free flour products, given its absence of gluten and excellent nutritional and organoleptic properties.

The proximate composition of biscuit formulations fortified with CP, AP, and BP is presented in Table 3. The enriched biscuits (B45RF50CF5CP, B45RF50CF5AP, B45RF50CF5BP) showed a slightly improved nutritional profile compared to the control (B50RF50CF), depending on the type of powder added. Moisture content decreased slightly from 7.28% in B50RF50CF to 7.01% in B45RF50CF5CP, likely due to the higher dietary fiber content from CF and the fruit powders [48]. Protein increased from 7.39% in the control to 7.50% in B45RF50CF5AP, lipids from 13.40% to a maximum of 15.08% (B45RF50CF5AP), ash from 3.32% to 3.42% (B45RF50CF5AP), and fiber from 5.58% to 6.46% (B45RF50CF5AP). Statistical analysis showed no significant differences (*p* > 0.05), but all enriched samples exhibited slightly higher values than the control. Sady and Sielicka-Różyńska [31] reported that adding 5–15% freeze-dried black chokeberry powder to functional biscuits increased ash, reduced lipids, and caused minimal changes in protein. Similarly, Raczkowska et al. [49] observed higher fiber and ash, with lower energy, in short crust pastries enriched with 10–50% chokeberry pomace. Lucas et al. [50] found increases in lipids, ash, and protein in extruded snacks with 2–6% freeze-dried açaí, while Šarić et al. [51] showed that replacing gluten-free flour with blueberry (28.2%) and raspberry (1.8%) pomace increased fiber without altering protein or carbohydrate content. These studies indicate that fruit powder addition generally improves the nutritional profile of baked products, particularly fiber, lipids, and ash. Overall, the addition of CP, AP, and BP improved the nutritional profile of the biscuits, particularly regarding fiber, lipids, and ash.

### 3.2. Physical Properties of Biscuit Formulations

After the chemical evaluation, biscuit samples were subjected to physical analysis. The physical properties (diameter, thickness, weight and spread ratio) of biscuits are shown in Table 4.

The results of the evaluation of the physical characteristics of the biscuits indicate that there were significant differences (*p* < 0.05) between the control sample (BRF) and the respective BCF samples B90RF10CF–B10RF90CF in terms of thickness, diameter, weight and spread ratio. The control biscuits (BRF) had a significantly larger diameter (60.10 mm) but smaller thickness (8.60 mm) compared to the BCF sample with 100% CF, which had a diameter of 53.42 mm and a thickness of 9.90 mm. In the case of B90RF10CF–B10RF90CF composite biscuit samples, the diameter decreased from 59.41 mm to 54.04 mm and the thickness increased from 8.73 mm to 9.77 mm proportional to the increase in the percentage of CF substitution compared to control biscuits. The same decreasing trend was recorded for the weight of biscuits with added CF compared to the control biscuits, which varied from 25.83 g in B90RF10CF to 25.14 g in B10RF90CF compared to 25.92 g in BRF. Statistical analysis of height and weight did not reveal significant differences among the analyzed samples (*p* > 0.05). However, the observed values of these two parameters were slightly higher and lower, respectively, compared to those of the control sample, B50RF50CF. The results of the biscuit spread ratio showed a significant decrease (*p* < 0.05) with increasing CF supplementation from 6.98 in BRF to 5.53 in B10RF90CF. Thus, with increasing CF concentration, the biscuit spread ratio gradually decreased from 6.80 in B90RF10CF to 5.53 in B10RF90CF. The reduction in the spread ratio of CF biscuits can be attributed to concomitant variations in diameter and thickness, highlighting the interdependence between dimensional parameters and spreading properties. The results obtained in the present study are consistent with those reported by Singh et al. [52] and Hegazy et al. [44], Demirkesen [5], and Torra et al. [6]. Composite flours may form aggregates with increased numbers of hydrophilic sites that compete for limited free water in cookie dough, thereby increasing dough viscosity and limiting cookie spread [53]. The results obtained are also consistent with the findings of other authors [54], according to whom, dietary proteins and fibers have more water-binding capacity. According to studies by Ganorkar and Jain [54], when flour components absorb large amounts of water, this reduces the amount of water available to dissolve the sugars in the dough and, therefore, the initial viscosity is higher and the biscuits stretch less during baking. Demirkesen [5] also found in his study a similar trend in biscuit sizes when RF was substituted for CF in biscuit dough formulas.

In the case of biscuit formulations fortified with CP, AP, and BP (Table 4), the evaluation of physical characteristics (thickness, diameter, weight, and spread ratio) did not reveal significant differences (*p* > 0.05) between the control sample (B50RF50CF) and the samples with added fruit powders (B45RF50CF5CP, B45RF50CF5AP, B45RF50CF5BP). The control sample had a diameter of 56.74 mm, a thickness of 9.25 mm, a weight of 25.49 g, and a spread ratio of 6.13. Statistical analysis did not reveal significant differences (*p* > 0.05) between biscuits supplemented with CP, AP, and BP and the control sample. Moreover, the addition of fruit powders did not significantly affect any of the measured physical parameters. Specifically, the diameter ranged from 56.40 mm (B45RF50CF5BP) to 56.49 mm (B45RF50CF5AP), thickness from 9.27 mm (B45RF50CF5AP) to 9.30 mm (B45RF50CF50BP), weight from 25.45 g (BRF50CF50BP) to 25.47 g (BRF50CF5CP), and spread ratio from 6.06 (B45RF50CF5BP) to 6.09 (B45RF50CF5AP). Consequently, biscuits fortified with CP, AP, and BP exhibited a lower spread ratio compared to the control biscuits. This result suggests that the inclusion of CP, AP, and BP tends to limit lateral expansion during baking without significantly altering the overall shape or physical integrity of the biscuits. The results obtained in the present study are consistent with those reported by Arun et al. [55], Choudhury et al. [56], and Saeed et al. [57].

### 3.3. Macro- and Microelement Profile of Composite Flours, Fruit Powders, and Biscuits

The macro- and micro-nutrient content of RF, CF, composite flours, CP, AP, BP, and biscuit formulations is presented in Table 5 and Table 6. Minerals are essential for metabolism and homeostasis, and deficiencies are linked to common disorders. Gluten-free diets often lack minerals such as calcium, magnesium, and iron, which are crucial for metabolic processes [2]. Including nutrient-rich foods is the most effective way to prevent deficiencies. CF is an important source of essential minerals, enhancing nutritional quality and supporting health benefits [8,15,58,59]. Replacing RF with CF increased K, P, Mg, Ca, Fe, Zn, Mn, and Cu in both composite flours and biscuits. Potassium and phosphorus were the most abundant minerals, while copper was the lowest. Potassium (K) content in CF was 2456.39 mg/kg versus 1050.47 mg/kg in RF and ranged from 1191.06 to 2315.80 mg/kg in composite flours, increasing with CF percentage. The K content in our CF sample (2456.39 mg/kg) was lower than reported in other studies (89.26–930 mg/100 g) [44,60]. Significant differences (*p* < 0.05) were observed in phosphorus (P) content among CF, RF, and composite flours. Phosphorus was 1220.25 mg/kg in CF, nearly double the 630.63 mg/kg in RF. In composite flours, P increased proportionally with CF substitution, from 689.59 mg/kg in 90RF10CF to 1161.20 mg/kg in 10RF90CF, consistent with literature values (124 mg/100 g) [44]. Magnesium (Mg) is essential for over 300 enzymatic reactions. CF contained significantly more Mg (840.67 mg/kg) than RF (636.08 mg/kg), and composite flours showed proportional increases with CF substitution, ranging from 656.54 mg/kg (90RF10CF) to 820.21 mg/kg (10RF90CF). Calcium (Ca) was also higher in CF (494.58 mg/kg) than RF (110.86 mg/kg), increasing in composites to 456.20 mg/kg in 10RF90CF, though lower than values reported elsewhere (500–599 mg/100 g) [44,61]. Iron (Fe), zinc (Zn), and manganese (Mn) in CF (32.07, 20.06, 18.18 mg/kg) were slightly higher than in RF (29.47, 14.34, 13.74 mg/kg), with composite flours reaching 31.80 mg/kg Fe, 19.50 mg/kg Zn, and 17.78 mg/kg Mn in 10RF90CF. Copper (Cu) levels were minimally affected by substitution, ranging from 3.078 mg/kg in RF to 5.476 mg/kg in 10RF90CF.

The macro- and microelement content of CP, AP, and BP differed significantly (*p* < 0.05). CP had the highest K (2514.21 mg/kg) and P (2326.87 mg/kg), AP the highest Mg (1560.82 mg/kg) and Ca (2210.73 mg/kg), and BP the highest Mn (26.46 mg/kg). Incorporation of these powders into composite flours substantially increased mineral content due to their high nutritional density. They are important natural sources of minerals (Fe, Zn, Mn, Mg, K, Ca), and their stability during processing helps maintain these levels. Bioactive compounds in the powders also enhance mineral bioavailability, improving the nutritional profile of the final product [24,26].

The mineral profile of biscuits with added CF is shown in Table 6. Higher macro- and microelement levels reflect the mineral richness of CF. K content was significantly higher in BCF (2192.83 mg/kg) than in BRF (889.26 mg/kg), with composite biscuits ranging from 2062.38 mg/kg in B10RF90CF to lower values in B90RF10CF. These findings agree with previous studies highlighting mineral enrichment of flour products through CF fortification [5,58,62]. According to WHO guidelines, the recommended daily potassium intake is 3510 mg/day; thus, 100 g of 50RF50CF biscuits provides about 4.39% of this requirement. Significant differences in phosphorus (P) were observed between BRF (389.26 mg/kg) and BCF (792.84 mg/kg), with composite biscuits ranging up to 752.41 mg/kg in B10RF90CF. P levels in our biscuits were lower (79.28 mg/100 g in BCF; 68.53 mg/100 g in B45RF50CF5CP) than reported in other studies (286 mg/100 g for 48% CF biscuits) [63]. Mg was higher in BCF (614.24 mg/kg) than in BRF (489.22 mg/kg), increasing to 600.88 mg/kg in B10RF90CF. A 100 g portion of B50RF50CF biscuits provides ~15.76% of the RDI for men and 18.39% for women (EFSA: 350 mg/day for men, 300 mg/day for women) [64]. Ca ranged from 79.14 mg/kg in BRF to 297.54 mg/kg in B10RF90CF, lower than values reported for 30% CF biscuits (450 mg/kg) [44] but higher than bread with 10% CF (125 mg/kg) [64]. Similar trends were observed in biscuits, with Fe ranging from 18.45 mg/kg in BRF to 21.75 mg/kg in B10RF90CF, Zn from 10.32 to 15.97 mg/kg, Mn from 8.42 to 12.20 mg/kg, and Cu from 1.23 to 3.01 mg/kg. These results are consistent with previous studies [5,58]. Analysis of biscuits with CF and 5% CP, AP, or BP showed a marked increase in mineral content, consistent with the high nutritional density and specific mineral composition of each powder. Specifically, B45RF50CF5CP showed the highest concentrations of K (1621.71 mg/kg), P (686.93 mg/kg), Fe (23.24 mg/kg), and Zn (19.56 mg/kg); B45RF50CF5AP exhibited the highest Mg (604.60 mg/kg) and Ca (306.45 mg/kg); and B45RF50CF5BP contained the highest Mn (11.41 mg/kg). These findings align with previous studies [63,64,65]. The lower mineral content in the baked biscuits compared to the composite flours (Table 5 vs. Table 6) reflects the composition of the biscuit recipe. Based on the proportions of ingredients used per 100 g of biscuits, the composite flours account for only a fraction of the total biscuit mass, while the remainder consists of butter, eggs, stevia, salt, and baking powder, which contribute to a lesser extent to the mineral content. Therefore, the minerals from the flours are distributed throughout the total biscuit matrix (i.e., effectively diluted), explaining the lower mineral values observed in the final baked product.

Partial replacement of RF with CF, CP, AP, or BP improves the mineral profile of fortified biscuits by increasing K, P, Mg, and Ca, while Fe, Zn, and Mn decrease and Cu remains stable. Overall, these ingredients act as functional mineral sources in flour products [5,58,62,63,64,65], providing an efficient means to enhance mineral intake and meet recommended daily allowances.

### 3.4. Phytochemical Profile and Antioxidant Activity of Composite Flours and Fruit Powders

The total phenolic content (TPC), total flavonoid content (TFC), and antioxidant activity (evaluated using FRAP and DPPH assays) were measured for composite flours and fruit powders, and the results are presented in Table 7 and Table 8. The results regarding the TPC of the analyzed composite flours and fruit powders are expressed as gallic acid equivalents (GAEs).

The results for TPC, TFC, and antioxidant activity (DPPH and FRAP) showed a significant dependence (*p* < 0.05) between RF-based controls and samples with different CF proportions. Adding CF increased the phytochemical content proportionally to its percentage. In flour samples, TPC (Table 7) was three times higher in CF (152.44 mg GAE/100 g d.s.) than in RF (59.10 mg GAE/100 g d.s.), with composite flours ranging from 67.54 mg GAE/100 g d.s. in 90RF10CF to 143.16 mg GAE/100 g d.s. in 10RF90CF. Literature values vary: Durazzo et al. [66] found 365.80 mg/100 g in aqueous extracts and 896.01 mg/100 g in residues, Shafi et al. [67] reported 4.25 mg GAE/1000 g, Conti et al. [68] 0.940 mg/g (traditional drying) and 2.720 mg/g (hot air drying at 70 °C), and Donno et al. [69] 55.37–87.47 mg GAE/100 g d.s. for two Italian CF varieties. These variations depend on variety, geographic origin, cultivation, and processing conditions. Based on numerous studies [6,10,43,44,59,68,70], CF is considered a valuable ingredient for innovative, enriched product formulations.

In this study, the phytochemical profile of biscuits was enhanced by substituting RF with 5% fruit powders (CP, AP, BP) rich in bioactive compounds. CP showed the highest TPC (5512.44 mg GAE/100 g d.s.), followed by BP (5482.71 mg GAE/100 g d.s.) and AP (5060.04 mg GAE/100 g d.s.). Incorporating these powders increased the TPC of composite flours, with the highest value in 45RF50CF5CP (377.41 mg GAE/100 g d.s.). These results reflect the enrichment of composite flours with bioactive compounds. Literature values for similar powders include Aronia: 4434 mg/100 g [71], Açaí berries: 4240 mg/100 g [26], and blueberries: 424.84–819.12 mg GAE/100 g fresh weight (f.w.) [72]. TPC in black blueberry powders ranged from 2494.40 to 3009.03 mg GAE/100 g d.s. [73] and 4951.01 mg GAE/100 g d.s. in commercial powder [71].

The total flavonoid content (TFC) followed the same increasing trend as TPC. Among the flours, the highest TFC was recorded in CF (123.57 mg QE/100 g d.s.) and the lowest in RF (44.46 mg QE/100 g d.s.). In composite flours, TFC increased significantly with CF content, from 51.69 mg QE/100 g d.s. in 90RF10CF to 115.76 mg QE/100 g d.s. in 10RF90CF. Partial replacement of RF with CF (0–90%) progressively enhanced the bioactive and antioxidant properties, resulting in 2.42- and 2.61-fold increases in TPC and TFC, respectively. Shafi et al. [67] reported similar TFC values (1.92 g QE/1000 g for CF) and proportional increases with CF addition in flour blends and baked products. Other reported TFC values include 7.77 mg CAE/100 g in sweet chestnuts [74] and 8.58 mg CAE/100 g f.w. in chestnut fruits [75]. The CP, AP, and BP powders exhibited high TFC, with the highest in CP (3966.48 mg QE/100 g d.s.), followed by BP (3711.44 mg QE/100 g d.s.) and AP (2442.03 mg QE/100 g d.s.). This significantly increased the total flavonoid content of the composite flours, with the highest value in the 45RF50CF5CP formulation (280.40 mg QE/100 g d.s.). Comparable literature values are 2831 mg QE/100 g d.s. for aronia [71], 607 mg/100 g d.s. for açaí [26], and 36.08 mg/100 g d.s. for blueberries [76].

The antioxidant activity of the samples was assessed using the 1,1-diphenyl-2-picrylhydrazyl (DPPH) radical scavenging assay and the ferric reducing antioxidant power (FRAP) assay. The FRAP values obtained for RF and CF raw materials, as well as for their mixtures at different proportions, are presented in Table 7. Among the raw materials, CF exhibited the highest antioxidant capacity (16.18 µM Fe^2+^/g d.s.), whereas RF showed the lowest value (8.09 µM Fe^2+^/g d.s.). This difference is attributed to the higher content of bioactive compounds with antioxidant properties in CF. Composite flour mixtures displayed intermediate values, ranging from 8.73 to 14.92 µM Fe^2+^/g d.s., depending on the proportion of CF in the blend. Statistical analysis indicated that these differences in antioxidant capacity were significant (*p* < 0.05). Antioxidant activity results, expressed as DPPH radical inhibition (%), are summarized in Table 8. The data in Table 8 highlight the significantly higher DPPH radical scavenging activity of CF compared to RF due to its elevated content of polyphenolic compounds, including flavonoids, phenolic acids, anthocyanins, and procyanidins. Consequently, CF exhibited a DPPH inhibition of 62.62%, roughly twice that of RF (28.14%), directly influencing the antioxidant capacity of the composite flours. The percentage of DPPH inhibition increased progressively with the substitution of RF by CF, reaching 59.94% in 10RF90CF. Statistically significant differences (*p* < 0.05) were observed among all composite and non-composite flours. Hegazy et al. [44] reported a DPPH scavenging activity of 37.50 µM/g for CF, compared to 4.17 µM/g for wheat flour. Composite flours containing 10–30% CF showed intermediate activities ranging from 7.75 to 13.81 µM/g. Shafi et al. [67] reported a similar antioxidant activity for CF (61.57%) and a comparable trend in chestnut-wheat flour composites. The potent antioxidant properties of CF are attributed to its complex phytochemical composition, which depends on cultivar, geographical origin, and processing conditions. In contrast, RF exhibited lower activity (11.23%), with a similar pattern observed in pasta formulations containing varying proportions of rice and CF [70].

### 3.5. Phytochemical Profile and Antioxidant Activity of Biscuit Formulations

**Figure 2:**
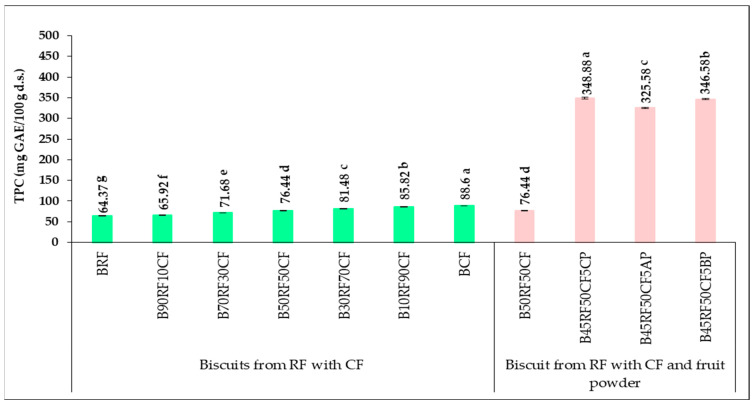
Total phenolic content (TPC) of the biscuit formulations. Set I (BRF, B90RF10CF, B70RF30CF, B50RF50CF, B30RF70CF, B10RF90CF, BCF): biscuits from rice flour with 0%, 10%, 30%, 50%, 70%, 90%, and 100% chestnut flour. Set II (B50RF50CF, B45RF50CF5CP, B45RF50CF5AP, B45RF50CF5BP): biscuits from rice flour with 50% CF plus 5% fruit powders—chokeberry powder, açaí powder and blueberry powder. Data are shown as mean ± standard deviation of three independent analyses. Values with different letters within the same set are significantly different (one-way ANOVA, *p* < 0.05), while those with the same letter are not significantly different (*p* > 0.05).

The TPC of the biscuit samples is shown in Figure 2 and expressed as mg GAE per 100 g. A similar increasing trend was observed in the composite biscuits, with TPC rising as the proportion of CF increased. The BRF sample (64.37 mg GAE/100 g d.s.) had a significantly lower TPC than the BCF sample (88.60 mg GAE/100 g d.s.). For biscuits made from composite flours, the lowest TPC was recorded in B90RF10CF (65.92 mg GAE/100 g d.s.), while the highest TPC was observed in B10RF90CF (85.82 mg GAE/100 g d.s.). These results indicate that total polyphenol content increased with higher CF levels in both composite flour and biscuit samples. The TPC of biscuits was lower than that of the corresponding flour samples, likely due to the high temperatures reached during baking [67,77]. A similar decrease in TPC in bread made from wheat flour during baking is attributed to the thermal instability of polyphenols [70]. The TPC in cookies reported by Shafi et al. [67] was 3.37 mg GAE/1000 g. In the same study, mixtures of wheat and chestnut flours in various proportions and cookies prepared from these composite flours showed a similar trend to that observed in the present work. Oniszczuk et al. [70] investigated the production of different types of gluten-free pasta from RF and CF in different proportions, reporting similar TPC values for RF (21.23 mg GAE/100 g d.s.) and a similar linear increase with higher CF proportions. Silav-Tuzlu and Tacer-Caba [14] studied biscuits made from chia seeds, CF, buckwheat, and potatoes and reported a TPC of 400.2 mg GAE/100 g d.s. and total antioxidant activity of 155.5 mg TE/100 g d.s.

Biscuit samples enriched with CP, AP, and BP showed significantly higher TPC values (Figure 2) than those made with only CF and RF. The highest TPC was recorded in B45RF50CF5CP (348.88 mg GAE/100 g d.s.), followed by B45RF50CF5BP (346.58 mg GAE/100 g d.s.), and the lowest in B45RF50CF5AP (325.58 mg GAE/100 g d.s.). These differences reflect the high polyphenol content of the added powders, with TPC values of 5512.44 mg GAE/100 g d.s. for CP, 5482.71 mg GAE/100 g d.s. for BP, and 5060.04 mg GAE/100 g d.s. for AP. The TPC increase in biscuits depended on the type of powder added (*p* < 0.05). Similar effects of fruit powders on TPC have been reported in other studies: papaya powder (15%) increased cake TPC from 0.01 to 0.6 mg/g [78], freeze-dried cranberry powder enriched cakes by 41–49% [79], and black chokeberry (15%) increased TPC in cookies to 6473.3 mg GAE/100 g d.s. [31]. Chestnut-fortified biscuits also showed 400.2 mg GAE/100 g d.s. [14].

**Figure 3:**
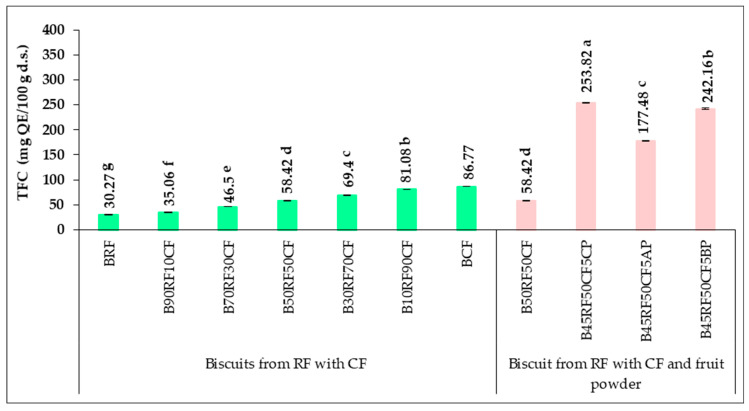
Total flavonoid content (TFC) of the biscuit formulations. Set I (BRF, B90RF10CF, B70RF30CF, B50RF50CF, B30RF70CF, B10RF90CF, BCF): biscuits from rice flour with 0%, 10%, 30%, 50%, 70%, 90%, and 100% chestnut flour. Set II (B50RF50CF, B45RF50CF5CP, B45RF50CF5AP, B45RF50CF5BP): biscuits from rice flour with 50% CF plus 5% fruit powders—chokeberry powder, açaí powder and blueberry powder. Data are shown as mean ± standard deviation of three independent analyses. Values with different letters within the same set are significantly different (one-way ANOVA, *p* < 0.05), while those with the same letter are not significantly different (*p* > 0.05).

The total flavonoid content (TFC) (Figure 3) followed a trend similar to TPC. TFC in biscuits was lower than in flours, ranging from 30.27 mg QE/100 g d.s. in BRF to 86.77 mg QE/100 g d.s. in BCF. The decrease in TFC is attributed to baking processes, likely due to oxidation caused by water, oxygen, and heat. Previous studies reported similar values and a proportional increase in TFC with higher CF content [67,80]. TFC increased proportionally with the percentage of CF, with statistically significant differences (*p* < 0.05). Biscuits enriched with CP, AP, and BP (B45RF50CF5CP, B45RF50CF5AP, B45RF50CF5BP) showed significantly higher TFC than those with only CF and RF, with the highest value in B45RF50CF5CP (253.82 mg QE/100 g d.s.), followed by B45RF50CF5BP (242.16 mg QE/100 g d.s.), and the lowest in B45RF50CF5AP (177.48 mg QE/100 g d.s.). This reflects the high flavonoid content of the powders: 3966.48 mg QE/100 g d.s. for CP, 3711.44 mg QE/100 g d.s. for BP, and 2442.03 mg QE/100 g d.s. for AP. Replacing 5% RF with CP, AP, or BP in B50RF50CF enhanced the functionality of the biscuits, resulting in 3.60- and 3.36-fold increases in TPC and TFC, respectively, compared to B50RF50CF.

Other authors reported TFC values of 376–7848 mg/100 g in chokeberry, approximately 600 mg/100 g in sea buckthorn, and around 285 mg/100 g in hawthorn, with lower values observed in baked biscuits due to heat [21]. Najjar et al. [81] found that adding 2.5–7.5% date powder to cookies increased TFC proportionally from 0.02 to 0.39% (*w*/*w*) compared to control cookies. These results demonstrate that CF, CP, AP, and BP enhance the bioactive profile of biscuits, with fruit powders having the strongest effect. The increases in TPC and TFC were dose-dependent and statistically significant (*p* < 0.05), confirming their contribution to improving the biscuits’ bioactive composition.

**Figure 4:**
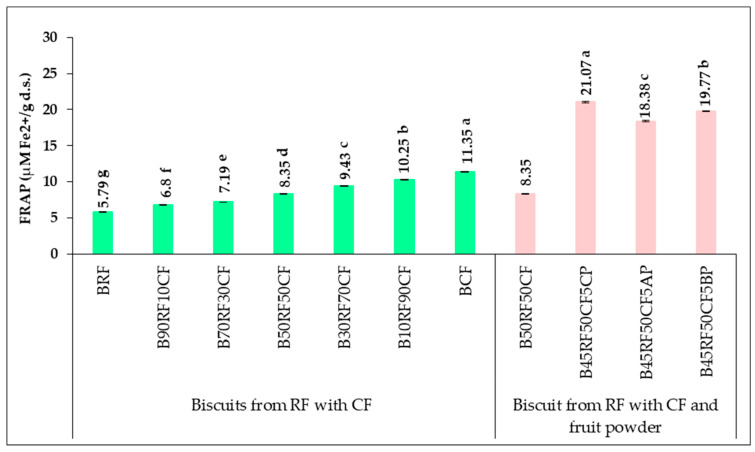
Antioxidant activity measured by FRAP in biscuit formulations. Set I (BRF, B90RF10CF, B70RF30CF, B50RF50CF, B30RF70CF, B10RF90CF, BCF): biscuits from rice flour with 0%, 10%, 30%, 50%, 70%, 90%, and 100% chestnut flour. Set II (B50RF50CF, B45RF50CF5CP, B45RF50CF5AP, B45RF50CF5BP): biscuits from rice flour with 50% CF plus 5% fruit powders—chokeberry powder, açaí powder and blueberry powder. Data are shown as mean ± standard deviation of three independent analyses. Values with different letters within the same set are significantly different (one-way ANOVA, *p* < 0.05), while those with the same letter are not significantly different (*p* > 0.05).

The antioxidant capacity of the biscuit samples (Figure 4, Table 9) mirrored the trends observed for TPC and TFC. Both FRAP and DPPH assays showed statistically significant increases (*p* < 0.05) in antioxidant activity with higher proportions of CF and fruit powders. 

According to the FRAP test (Figure 4), the BRF sample showed the lowest antioxidant capacity, of 5.79 µM Fe^2+^/g d.s.Gradual supplementation with CF in BRF increased the FRAP value to 10.25 µM Fe^2+^/g d.s. in sample B10RF90CF, representing a 1.78-fold increase. Furthermore, replacing 5% of RF with CP, AP, or BP in the B50RF50CF formula led to a further improvement, with FRAP reaching 21.07 µM Fe^2+^/g d.s. in B45RF50CF5CP, 2.52-fold more than in B50RF50CF.

The results obtained for DPPH and FRAP assays clearly demonstrate a strong correlation between CF enrichment and enhanced antioxidant capacity in biscuit formulations. The DPPH inhibition (Table 9) increased from 27.54% in BRF to 59.29% in B10RF90CF (2.15-fold) and 62.45% in BCF (2.27-fold), while FRAP values showed a parallel trend. This proportional enhancement confirms that CF contributes significantly to the antioxidant potential of composite products.

The incorporation of CP, AP, and BP further intensified this effect, with DPPH activity ranging between 48.57% for B45RF50CF5AP and 50.36% for B45RF50CF5CP. These findings suggest a synergistic interaction between CF and fruit-derived powders, which together supply a broader spectrum of phenolic compounds and enhance the redox potential of the matrix.

Similar tendencies have been previously reported by Shafi et al. [67], Silav-Tuzlu and Tacer-Caba [14], and Kossyva et al. [16], emphasizing the beneficial role of CF in antioxidant-enriched bakery products. The marked improvement in antioxidant activity can be attributed to the high concentration of polyphenols, flavonoids, and anthocyanins present in CF and the added fruit powders. The observed dose-dependent increase in both TPC and TFC aligns with previous reports indicating a strong correlation between TPC and antioxidant activity [82].

Moreover, the persistence of high antioxidant values after baking indicates that a considerable portion of these bioactive compounds remains stable under thermal processing, thereby maintaining their functional properties in the final product. Overall, these results reinforce the potential of CF and fruit powders (CP, AP, BP) as effective functional ingredients capable of improving the bioactive composition and oxidative stability of bakery formulations. Their combined application not only enhances nutritional and antioxidant properties but also supports the development of innovative, health-oriented cereal-based foods with significant functional and commercial potential.

### 3.6. Characterization of Flours, Fruit Powders and Biscuit Formulations Using Fourier Transform Infrared Spectroscopy (FTIR)

**Figure 5:**
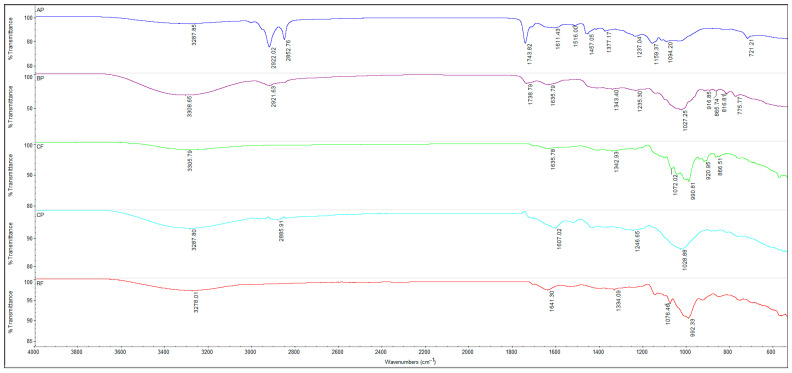
FTIR spectra of flours and fruit powders, spectral range of 4000–600 cm^−1^, 32 scans at 4 cm^−1^ resolution. RF: rice flour; CF: chestnut flour; CP: chokeberry powder; AP: açaí powder; BP: blueberry powder. Line colors: RF—red, CF—green, CP—light blue, AP—blue, BP—mauve.

Fourier Transform Infrared (FTIR) spectroscopy was used to analyze the functional composition of RF, CF, the fruit powders (CP, AP, BP), and the biscuit formulations (BRF, BCF, B45RF50CF5CP, B45RF50CF5AP, B45RF50CF5BP), in order to highlight molecular interactions and structural changes resulting from the substitution of RF with CF and fruit powders (Figure 5). The spectral interpretation provided essential insights into the chemical composition and matrix interactions of the analyzed samples. The FTIR spectrum of RF (Figure 5) exhibited a distinct absorption band at 3728 cm^−1^, corresponding to the stretching vibrations of free –OH groups from polysaccharides (starch, cellulose) and weakly bound water. The band at 2885 cm^−1^ was assigned to the symmetric C–H stretching vibrations of methylene groups (–CH_2_–), mainly linked to starch and, to a lesser extent, lipid and protein fractions. The absorption band at 1641 cm^−1^ corresponded to C=O stretching vibrations (Amide I), confirming the presence of proteins in the RF matrix. In the fingerprint region (1500–600 cm^−1^), bands at 1076 cm^−1^ and 992 cm^−1^ were attributed to C–O and C–O–C stretching vibrations characteristic of polysaccharides, consistent with the starch structure of RF. These results align with previous reports [83,84,85,86,87].

The FTIR spectrum of CF (Figure 5) revealed a strong absorption band at 3305 cm^−1^, corresponding to O–H stretching vibrations typical of polysaccharides and phenolic compounds. This result aligns with reported hydroxyl bands observed between 3300–3500 cm^−1^ in plant-based flours. The absorption at 1635 cm^−1^ was attributed to C=O stretching of amide I bonds, confirming the presence of proteins, while the band at 1342 cm^−1^ (amide III) was assigned to N–H bending and C–N stretching of peptide bonds, providing complementary information on protein conformation. Additional peaks detected in the 1076–992 cm^−1^ region correspond to C–O and C–O–C stretching vibrations of polysaccharides and starch, confirming the dominance of carbohydrate structures. These findings demonstrate the coexistence of protein and polysaccharide fractions in CF, which play a crucial role in its hydration and rheological properties. The results are consistent with previous studies [82,83].

The FTIR spectra of CP, AP, and BP showed absorption bands at 3280–3300 cm^−1^, corresponding to O–H stretching of polysaccharides, phenolic compounds, and residual water, reflecting hydrogen bonding and intermolecular interactions that influence functional properties and rheology. Bands at 2852–2922 cm^−1^ were attributed to C–H stretching of –CH_2_ and –CH_3_ groups. In BP, the 1740 cm^−1^ band indicated C=O stretching of phenolic esters and organic acids, while signals at 1600–1650 cm^−1^ corresponded to aromatic C=C and flavonoid C=O stretching, reflecting significant polyphenol and flavonoid content. Bands at 1000–1100 cm^−1^ were assigned to C–O stretching of polysaccharides and hydroxyl groups from phenols and sugars, contributing to solubility and hydration. Partial replacement of RF with CF, CP, AP, and BP induces progressive changes in the FTIR spectra, revealing a complex matrix enriched with polysaccharides, polyphenols, and proteins. These components act synergistically to enhance hydration behavior, structural stability, rheological properties, and antioxidant capacity, confirming their functional potential in food formulations [83,88].

**Figure 6:**
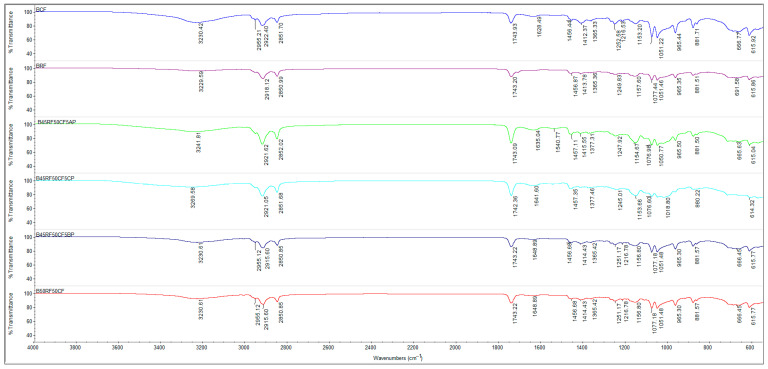
FTIR spectra of biscuit formulations, spectral range of 4000–600 cm^−1^, 32 scans at 4 cm^−1^ resolution. BRF, B50RF50CF, BCF: biscuits from rice flour with 0%, 50% and 100% chestnut flour; B45RF50CF5CP, B45RF50CF5AP, B45RF50CF5BP: biscuits from rice flour with 50% CF plus 5% fruit powders—chokeberry powder, açaí powder and blueberry powder. Line colors: B50RF50CF—red, B45RF50CF5BP—dark blue, B45RF50CF5CP—light blue, B45RF50CF5AP—green, BRF—mauve and BCF—blue.

The FTIR spectra of the biscuit formulations (BRF, BCF, B45RF50CF5CP, B45RF50CF5AP, B45RF50CF5BP) showed clear spectral changes after partial substitution of RF with CF and fruit powders (CP, AP, BP) (Figure 6). The broadened band at 3220–3270 cm^−1^ reflects enhanced hydrogen bonding among polysaccharides, polyphenols, and water, supporting improved hydration and matrix cohesion [83]. Increased intensity of bands at 2850 cm^−1^, 1740 cm^−1^, and 1620–1650 cm^−1^ indicates stronger interactions among starch, lipids, proteins [85], and bioactive compounds, enhancing structural stability and antioxidant potential [82,88]. Amide I and III bands (1620–1650 and 1360–1380 cm^−1^) reflect protein network formation [86], while bands at 1410–1460 cm^−1^ show CH_2_/CH_3_ bending, contributing to biscuit texture and stability [83]. In the fingerprint region (1150–1250 cm^−1^), enhanced intensity confirms carbohydrate and polyphenol structures, influencing solubility, hydration, and antioxidant activity. Bands at 960 and 880 cm^−1^ correspond to carbohydrate and starch vibrations, reflecting their structural behavior. The FTIR spectra of biscuits enriched with CF and fruit powders indicate complex synergistic interactions between polysaccharides, proteins, and polyphenols. These interactions lead to a reorganization of the food matrix at the molecular level, promoting more efficient hydration, enhanced internal structural stability, and improved texture of the biscuits. Furthermore, polyphenol–protein interactions contribute to increased bioactivity, including antioxidant potential. Thus, FTIR analysis provides a detailed insight into how unconventional ingredients simultaneously influence the physicochemical and functional properties of fortified bakery products.

### 3.7. Small- and Wide-Angle X-Ray Scattering (SAXS/WAXS) Analysis of Flours, Fruit Powders and Biscuit Formulations

**Figure 7:**
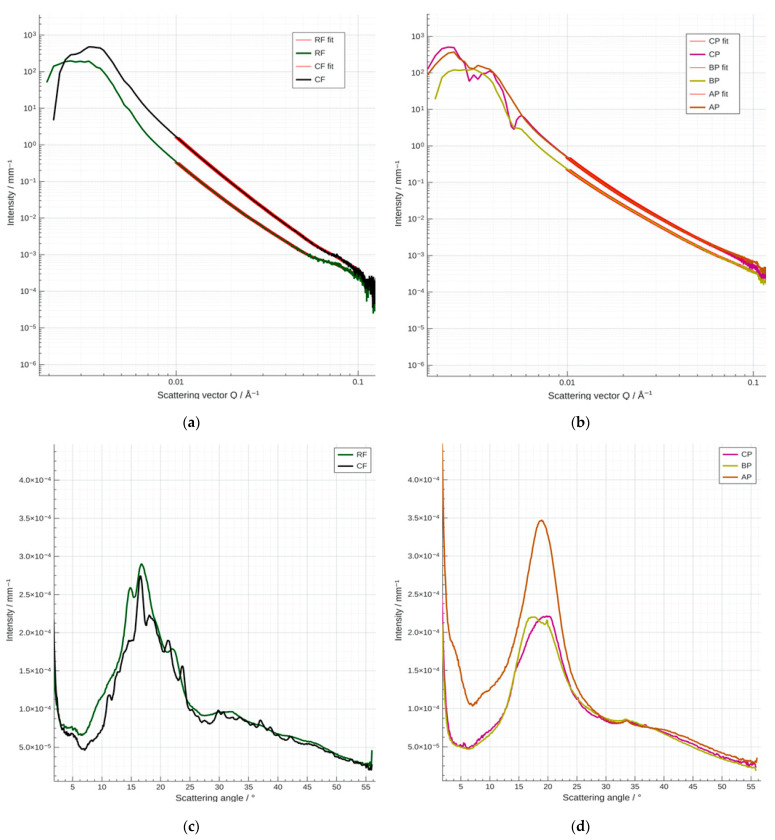
Small- and Wide-Angle X-ray Scattering (SAXS/WAXS) analysis of flours and fruit powders: (**a**) SAXS analysis of RF and CF; (**b**) SAXS analysis of CP, AP and BP; (**c**) WAXS analysis of RF and CF; (**d**) WAXS analysis of CP, AP and BP. RF: rice flour; CF: chestnut flour; CP: chokeberry powder; AP: açaí powder; BP: blueberry powder. Line colors: RF—green, CF—black, CP—pink, AP—orange, BP—light green.

**Figure 8:**
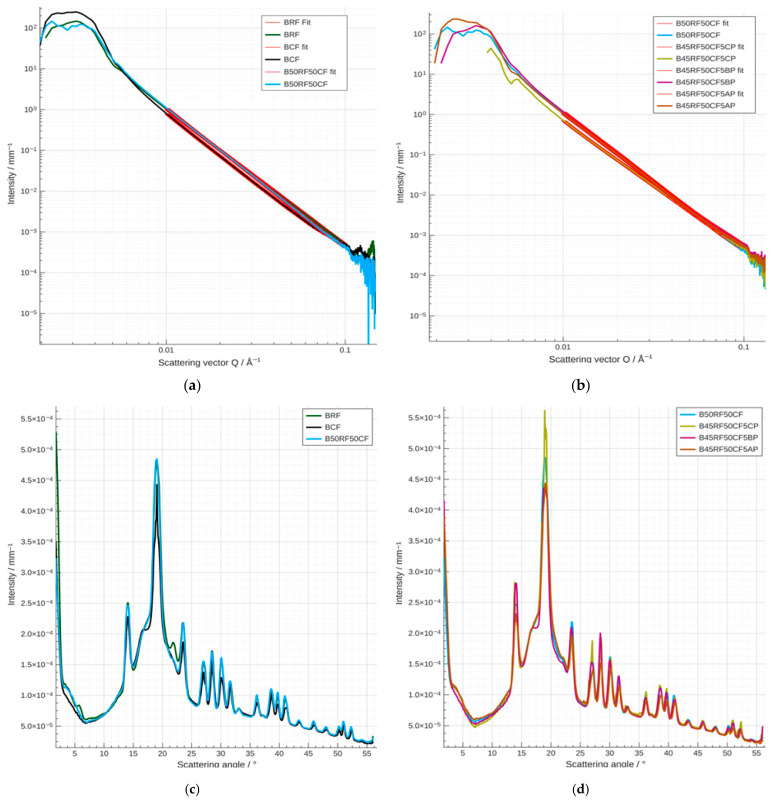
Small- and Wide-Angle X-ray Scattering (SAXS/WAXS) analysis of biscuit samples: (**a**) SAXS analysis of BRF, B50RF50CF and BCF; (**b**) SAXS analysis of B50RF50CF, B45RF50CF5CP, B45RF50CF5AP and B45RF50CF5BP; (**c**) WAXS analysis of BRF, B50RF50CF and BCF; (**d**) WAXS analysis of B50RF50CF, B45RF50CF5CP, B45RF50CF5AP and B45RF50CF5BP. BRF, B50RF50CF, BCF: biscuits from rice flour with 0%, 50% and 100% chestnut flour; B50RF50CF: biscuits from rice flour with 50% CF; B45RF50CF5CP, B45RF50CF5AP, B45RF50CF5BP: biscuits from rice flour with 50% CF plus 5% fruit powders—chokeberry powder, açaí powder and blueberry powder. Line colors: BRF—green, BCF—black, B50RF50CF—light blue, B45RF50CF5CP—light green, B45RF50CF5AP—orange, B45RF50CF5BP—pink.

SAXS/WAXS analysis provides important information on the crystalline organization of starch granules and proteins in RF, influenced by the addition of CF and fruit powders (CP, AP, and BP) (Table 10, Figure 7 and Figure 8). The results highlight the predominance of the type-A crystalline phase of starch in RF, accompanied by amorphous regions characteristic of the granules [89], while interactions with proteins and bioactive components from CF and fruit powders altered the intensity and width of the bands, suggesting a slight disruption of the crystalline structure. SAXS region analysis revealed significant structural differences between RF and CF. The smaller characteristic median (Table 10) size of RF (850.25 nm) compared to CF (912.98 nm), along with a much lower “surface-equivalent” value (40.31 nm versus 134.13 nm), indicates a compact structure of the RF fractions and, respectively, the tendency of CF to form larger aggregates with irregular structure [90,91]. CP showed a fine and uniform structure (median = 821.78 nm; surface-equivalent = 35.68 nm), AP displayed a less uniform structure (median = 784.48 nm; surface-equivalent = 25.69 nm), and BP exhibited a very fine structure (median = 757.67 nm; surface-equivalent = 24.31 nm). SAXS analysis (Table 10) revealed a median particle size of 866.67 nm and a surface-equivalent of 71.49 nm for biscuits made from 100% RF (BRF), indicating larger aggregates and partial disruption of the starch semicrystalline order [89]. Biscuits from the 50% RF + 50% CF blend exhibited higher values (median = 884.21 nm; surface-equivalent = 87.44 nm), suggesting a partially ordered structure and moderately firm texture due to partial starch preservation and the formation of intermediate aggregates.

Biscuits made exclusively from CF exhibited large, heterogeneous granules (median = 864.74 nm; surface-equivalent = 55.36 nm), with fragmentation increasing the amorphous phase and producing denser products with reduced porosity, in agreement with literature reports [92].

Structural differences between RF and CF significantly affect their behavior during baking. Fine, uniform granules in RF favor stable lamellar organization and high crystallinity, resulting in crispy, airy biscuits [93]. Conversely, the larger, more heterogeneous granules in CF disrupt this organization, yielding denser biscuits with a softer texture [94]. Intermediate flour blends allow modulation of mechanical properties, offering opportunities to enhance gluten-free biscuits.

The influence of functional fruit powders (CP, AP, BP) on gluten-free biscuits was assessed by incorporating 5% into the base formulation (50% RF + 50% CF). The powders exhibited distinct particle sizes and heterogeneities, reflecting their high phenolic, fiber, and sugar contents. Substituting RF with fruit powders altered SAXS/WAXS parameters: CP biscuits (median = 847.38 nm; surface-equivalent = 52.85 nm) preserved lamellar organization, stabilized by polyphenols; AP biscuits (median = 867.71 nm; surface-equivalent = 65.59 nm) showed slight structural disruption from lipid–amylopectin interactions; BP biscuits (median = 872.78 nm; surface-equivalent = 68.91 nm) exhibited the greatest disruption due to amorphous components and sugars interfering with starch retrogradation [95]. Textural outcomes followed this trend: CP and AP biscuits remained crispy, while BP biscuits were softer. Overall, fruit powder addition allows modulation of structural and textural properties, providing a strategy to improve the quality of gluten-free biscuit formulations. The SAXS analysis results (Figure 7a,b) revealed clear structural differences between flours and fruit powders. The flours (RF, CF) showed high scattering intensity in the Q < 0.01 Å^−1^ region, characteristic of starch granules. In contrast, the fruit powders (CP, AP, BP) exhibited lower intensity and smoother curves, indicating a predominantly amorphous and heterogeneous morphology. The scattering curves of the biscuits (Figure 8a,b) confirmed that the structural morphology was dominated by the flour matrix (RF/CF), as all curves nearly overlapped. However, biscuits containing 5% fruit powder (e.g., B45RF50CF5CP) showed a slight decrease in low-Q intensity (Q < 0.01 Å^−1^) compared to the control sample (B50RF50CF), reflecting minor perturbation of the flour aggregate organization and a reduction in electron density contrast.

WAXS analysis reveals the crystalline order of flours and fruit powders and highlights structural changes induced by the addition of CF, CP, AP, and BP (Figure 7c,d and Figure 8c,d). Variations in peak intensity and width indicate that these ingredients modulate starch crystallinity and affect the starch–protein matrix, influencing functional and textural properties such as cohesion and water retention [94].

Scattering angle peaks (2θ) from the WAXS analysis of flours and fruit powders are reported in Table S1, whereas Table S2 contains the corresponding data for the biscuit formulations.

In the amorphous region (scattering angle 2θ = 5–12°), RF and CF show high intensity, reflecting amorphous starch and protein fractions, while AP exhibits very high intensity at low scattering angles (2θ = 5°), suggesting large molecular complexes or fiber/pectin aggregates. CP and BP show lower intensity in this region, consistent with a predominantly amorphous morphology [95]. In the starch crystallinity region (scattering angle 2θ = 15–24°), RF and CF show sharp peaks at 15°, 17°, and 23° (A-type or A/B-type starch), whereas CP and BP show peak flattening and reduced intensity, indicating decreased relative crystallinity. These observations confirm that the balance between amorphous and crystalline fractions is influenced by sugars, lipids, polyphenols, and fiber content in each powder [94,96].

SAXS/WAXS analysis of biscuit formulations reveals the balance between crystalline and amorphous phases within the starch–protein matrix. While diffraction patterns largely overlap, differences are evident. In the amorphous region (2θ = 5–12°), BRF and BCF show moderate intensity, reflecting amorphous starch and protein fractions, which promote a lighter, crispier texture [89,90]. B45RF50CF5CP and B45RF50CF5AP exhibit higher amorphous intensity, suggesting sugars and lipids from fruit powders act as plasticizers, whereas B45RF50CF5BP shows a lower amorphous signal, indicating more ordered crystalline regions. In the starch crystallinity region (2θ = 15–24°), baking-induced starch retrogradation dominates. Fruit powder-fortified biscuits show decreased crystallinity or peak flattening due to interference of amorphous components with starch chain packing [7,97], resulting in denser products with reduced porosity [8,98]. Polyphenols in CP may partially stabilize the structure. SAXS has emerged as a powerful technique for examining the structure, conformation, interactions, and reaction dynamics of biomolecules, providing valuable molecular-level insights into the relationship between structure and function in various food processing applications [99]. The incorporation of CF and fruit powders (CP, AP, BP) into biscuit formulations significantly alters the starch–protein matrix, reducing starch crystallinity and increasing the amorphous fraction. These structural modifications, evidenced by FTIR and SAXS/WAXS analyses, improve hydration, solubility, digestibility, and textural flexibility, while enhancing the bioactive and antioxidant potential of the biscuits. Overall, the results confirm the functional value of CF and fruit powders for the development of nutritionally enriched gluten-free bakery products.

## 4. Conclusions

This study demonstrates the strong potential of chestnut flour (CF) and selected fruit powders to enhance the nutritional, bioactive, and technological quality of gluten-free biscuits. Substituting rice flour with CF markedly increased fiber, protein, lipid, and essential mineral contents, alongside important improvements in antioxidant attributes. The incorporation of 5% fruit powders provided an additional functional enhancement, with chokeberry producing the highest antioxidant intensification, underscoring the complementary effects of these bioactive-rich ingredients. From a technological perspective, biscuit structure was largely preserved even at high CF inclusion levels, with only minor decreases in diameter, weight, and spread ratio. Fruit powders slightly reduced lateral expansion during baking but did not compromise overall integrity or textural quality. The formulation combining 50% CF with 5% fruit powder achieved the most favorable balance, integrating improved nutritional and bioactive properties with acceptable structural performance. Advanced structural analyses (SAXS/WAXS and FTIR) revealed formulation-dependent modifications in starch crystallinity and protein network organization, offering molecular support for the observed enhancements in texture, gelatinization behavior, and system stability. These results confirm that unconventional flours and fruit-derived powders can be successfully incorporated into gluten-free matrices, generating innovative bakery products with significant functional benefits. Overall, the findings contribute to a comprehensive framework linking ingredient composition to matrix development, molecular interactions, and functional performance in gluten-free systems. Future research will extend this work by incorporating structured sensory evaluation conducted with trained human panelists to assess texture, crispness, color perception, and overall acceptability, thereby connecting perceptible product attributes with the structural insights obtained from FTIR and SAXS/WAXS analyses. In addition, multivariate statistical approaches will be applied to the full set of physico-chemical, nutritional, bioactive, and structural parameters to identify key factors driving formulation quality and guide optimization of ingredient combinations. These developments, together with detailed polyphenol profiling, shelf-life monitoring, and evaluation of structural and textural evolution during storage, will support the continued advancement of functional gluten-free biscuits.

## Figures and Tables

**Table 1:** Formulations of gluten-free rice flour biscuits: control sample and fortified variants with chestnut flour or with chestnut flour plus fruit powder.

Ingredients (g)	Gluten-Free Biscuit Formulations										
			Set I						Set II		
	BRF	B90RF10CF	B70RF30CF	B50RF50CF	B30RF70CF	B10RF90CF	BCF	B50RF50CF	B45RF50CF5CP	B45RF50CF5AP	B45RF50CF5BP
RF	160	144	112	80	48	16	-	80	72	72	72
CF	-	16	48	80	112	144	160	80	80	80	80
Stevia	100	100	100	100	100	100	100	100	100	100	100
Butter	110	110	110	110	110	110	110	110	110	110	110
Egg	100	100	100	100	100	100	100	100	100	100	100
Salt	2	2	2	2	2	2	2	2	2	2	2
Baking powder	8	8	8	8	8	8	8	8	8	8	8
CP	-	-	-	-	-	-	-	-	8	-	-
AP	-	-	-	-	-	-	-	-	-	8	-
BP	-	-	-	-	-	-	-	-	-	-	8

RF: rice flour; CF: chestnut flour; CP: chokeberry powder; AP: açaí powder; BP: blueberry powder. Set I (BRF, B90RF10CF, B70RF30CF, B50RF50CF, B30RF70CF, B10RF90CF, BCF): biscuits from rice flour with 0%, 10%, 30%, 50%, 70%, 90%, and 100% chestnut flour. Set II (B50RF50CF, B45RF50CF5CP, B45RF50CF5AP, B45RF50CF5BP): biscuits from rice flour with 50% CF plus 5% fruit powders—chokeberry powder, açaí powder and blueberry powder.

**Table 2:** Proximate composition and energy value of rice flour, chestnut flour, fruit powders and composite flours.

	Chemical Parameters						Energy Value (kcal/100 g)
Sample	Moisture (%)	Protein (%)	Lipids (%)	Ash (%)	CRB (%)	Crude Fiber (%)	
RF	10.33 ± 0.16 ^a^	5.75 ± 0.03 ^f^	1.65 ± 0.26 ^e^	2.57 ± 0.12 ^a^	79.70 ± 0.22 ^a^	2.65 ± 0.11 ^g^	356.61 ± 0.11 ^g^
90RF10CF	10.04 ± 0.10 ^b^	5.85 ± 0.02 ^e^	1.86 ± 0.22 ^e^	2.59 ± 0.09 ^a^	79.66 ± 0.24 ^a^	3.31 ± 0.14 ^f^	358.76 ± 0.13 ^f^
70RF30CF	9.46 ± 0.16 ^c^	6.04 ± 0.05 ^d^	2.29 ± 0.19 ^d^	2.64 ± 0.08 ^a^	79.57 ± 0.22 ^a^	4.62 ± 0.13 ^e^	363.06 ± 0.12 ^e^
50RF50CF	8.88 ± 0.32 ^d^	6.23 ± 0.04 ^c^	2.72 ± 0.21^c^	2.68 ± 0.10 ^a^	79.49 ± 0.23 ^a^	5.94 ± 0.15 ^d^	367.37 ± 0.10 ^d^
30RF70CF	8.29 ± 0.14 ^e^	6.42 ± 0.03 ^b^	3.15 ± 0.14 ^b^	2.72 ± 0.10 ^a^	79.41 ± 0.25 ^a^	7.25 ± 0.08 ^c^	371.67 ± 0.11 ^c^
10RF90CF	7.71 ± 0.16 ^f^	6.62 ± 0.15 ^a^	3.58 ± 0.27 ^a^	2.77 ± 0.07 ^a^	79.32 ± 0.23 ^a^	8.57 ± 0.12 ^b^	375.98 ± 0.14 ^b^
CF	7.42 ± 0.12 ^g^	6.71 ± 0.12 ^a^	3.79 ± 0.20 ^a^	2.79 ± 0.10 ^a^	79.28 ± 0.22 ^a^	9.23 ± 0.15 ^a^	378.13 ± 0.13 ^a^
CP	2.86 ± 0.17 ^c^	6.66 ± 0.17 ^c^	4.45 ± 0.11 ^b^	2.46 ± 0.08 ^c^	83.57 ± 0.12 ^a^	18.22 ± 0.14 ^b^	400.98 ± 0.04 ^b^
AP	5.56 ± 0.13 ^b^	9.02 ± 0.15 ^a^	45.11 ± 0.19 ^a^	4.87 ± 0.11 ^a^	35.44 ± 0.11 ^c^	20.30 ± 0.15 ^a^	583.83 ± 0.09 ^a^
BP	7.90 ± 0.14 ^a^	7.28 ± 0.16 ^b^	4.13 ± 0.18 ^c^	4.03 ± 0.14 ^b^	76.65 ± 0.10 ^b^	14.14 ± 0.12 ^c^	372.93 ± 0.05 ^c^
50RF50CF	8.88 ± 0.32 ^a^	6.23 ± 0.04 ^a^	2.72 ± 0.21^b^	2.68 ± 0.10 ^a^	79.49 ± 0.23 ^a^	5.94 ± 0.15 ^b^	367.37 ± 0.10 ^d^
45RF50CF5CP	8.50 ± 0.30 ^a^	6.28 ± 0.14 ^a^	2.86 ± 0.29 ^b^	2.72 ± 0.09 ^a^	79.68 ± 0.34 ^a^	6.72 ± 0.13 ^a^	369.59 ± 0.12 ^b^
45RF50CF5AP	8.64 ± 0.29 ^a^	6.40 ± 0.15 ^a^	4.89 ± 0.27 ^a^	2.80 ± 0.08 ^a^	77.28 ± 0.32 ^b^	6.82 ± 0.12 ^a^	378.73 ± 0.12 ^a^
45RF50CF5BP	8.75 ± 0.31 ^a^	6.31 ± 0.12 ^a^	2.84 ± 0.30 ^b^	2.75 ± 0.11 ^a^	79.34 ± 0.33 ^a^	6.51 ± 0.13 ^a^	368.19 ± 0.13 ^c^

CRB: carbohydrates; RF: rice flour; CF: chestnut flour; CP: chokeberry powder; AP: açaí powder; BP: blueberry powder. Set I (RF, 90RF10CF, 70RF30CF, 50RF50CF, 30RF70CF, 10RF90CF, and CF): rice flour with 0%, 10%, 30%, 50%, 70%, 90%, and 100% chestnut flour. Set II (50RF50CF, 45RF50CF5CP, 45RF50CF5AP, and 45RF50CF5BP): rice flour with 50% CF plus 5% fruit powders—chokeberry powder, açaí powder, and blueberry powder. Data are presented as mean ± standard deviation of three independent analyses. Values with different letters within the same column are significantly different (one-way ANOVA, *p* < 0.05), while those with the same letter are not significantly different (*p* > 0.05).

**Table 3:** Proximate composition of biscuits (control samples and fortified formulations).

Sample	Chemical Parameters						
	Moisture (%)	Protein (%)	Lipids (%)	Ash (%)	Total Carbohydrates (%)	Crude Fiber (%)	Energy Value (kcal/100 g)
BRF	8.39 ± 0.21 ^a^	6.88 ± 0.16^c^	11.49 ± 0.32 ^f^	2.89 ± 0.12 ^e^	70.39 ± 0.02 ^a^	2.73 ± 0.11 ^b^	412.36 ± 0.11 ^g^
B90RF10CF	8.16 ± 0.14 ^a^	6.96 ± 0.06 ^c^	11.87 ± 0.06 ^e^	2.97 ± 0.10 ^e^	70.04 ± 0.06 ^b^	3.30 ± 0.14 ^c^	414.82 ± 0.17 ^f^
B70RF30CF	7.72 ± 0.19 ^b^	7.17 ± 0.14 ^b^	12.64 ± 0.19 ^d^	3.15 ± 0.07 ^d^	69.33 ± 0.10 ^c^	4.44 ± 0.16 ^d^	419.73 ± 0.12 ^e^
B50RF50CF	7.28 ± 0.18 ^c^	7.39 ± 0.17 ^b^	13.40 ± 0.14 ^c^	3.32 ± 0.05 ^c^	68.62 ± 0.08 ^d^	5.58 ± 0.12 ^e^	424.64 ± 0.09 ^d^
B30RF70CF	6.83 ± 0.14 ^d^	7.60 ± 0.11^b^	14.17 ± 0.12 ^b^	3.49 ± 0.02 ^c^	67.90 ± 0.04 ^e^	6.72 ± 0.13 ^f^	429.55 ± 0.12 ^c^
B10RF90CF	6.39 ± 0.15 ^e^	7.82 ± 0.10 ^a^	14.93 ± 0.21 ^a^	3.66 ± 0.09 ^a^	67.20 ± 0.02 ^f^	7.87 ± 0.12 ^g^	434.46 ± 0.11 ^b^
BCF	6.16 ± 0.12 ^e^	7.93 ± 0.15 ^a^	15.32 ± 0.23 ^a^	3.75 ± 0.13 ^a^	66.84 ± 0.09 ^g^	8.44 ± 0.13 ^a^	436.92 ± 0.16 ^a^
B50RF50CF	7.28 ± 0.18 ^a^	7.39 ± 0.17 ^a^	13.40 ± 0.14 ^b^	3.32 ± 0.05 ^a^	68.62 ± 0.08 ^c^	5.58 ± 0.12 ^b^	424.64 ± 0.21 ^b^
B45RF50CF5CP	7.01 ± 0.15 ^a^	7.43 ± 0.26 ^a^	13.45 ± 0.07 ^b^	3.34 ± 0.05 ^a^	69.27 ± 0.16 ^a^	6.36 ± 0.17 ^a^	424.07 ± 0.20 ^c^
B45RF50CF5AP	7.13 ± 0.17 ^a^	7.50 ± 0.16 ^a^	15.08 ± 0.05 ^a^	3.42 ± 0.01 ^a^	66.87 ± 0.18 ^d^	6.46 ± 0.14 ^a^	433.21 ± 0.24 ^a^
B45RF50CF5BP	7.25 ± 0.12 ^a^	7.46 ± 0.23 ^a^	13.50 ± 0.06 ^b^	3.38 ± 0.07 ^a^	68.93 ± 0.16 ^b^	6.15 ± 0.09 ^a^	422.67 ± 0.19 ^d^

Biscuit formulations: Set I (BRF, B90RF10CF, B70RF30CF, B50RF50CF, B30RF70CF, B10RF90CF, BCF): biscuits from rice flour with 0%, 10%, 30%, 50%, 70%, 90%, and 100% chestnut flour. Set II (B50RF50CF, B45RF50CF5CP, B45RF50CF5AP, B45RF50CF5BP): biscuits from rice flour with 50% CF plus 5% fruit powders—chokeberry powder, açaí powder and blueberry powder. Data are presented as mean ± standard deviation of three independent analyses. Values with different letters within the same column are significantly different (one-way ANOVA, *p* < 0.05), while those with the same letter are not significantly different (*p* > 0.05).

**Table 4:** Physical properties of biscuits (control samples and fortified formulations).

Sample	Physical Properties			
	Diameter (mm)	Thickness (mm)	Weight (g)	Spread Ratio
BRF	60.10 ± 0.19 ^a^	8.60 ± 0.17 ^a^	25.92 ± 0.15 ^a^	6.98 ± 0.03 ^a^
B90RF10CF	59.41 ± 0.18 ^b^	8.73 ± 0.12 ^a^	25.83 ± 0.18 ^a^	6.80 ± 0.02 ^b^
B70RF30CF	58.13 ± 0.09 ^c^	8.99 ± 0.15 ^a^	25.66 ± 0.17 ^a^	6.46 ± 0.05 ^c^
B50RF50CF	56.74 ± 0.13 ^d^	9.25 ± 0.16^a^	25.49 ± 0.11 ^a^	6.13 ± 0.06 ^d^
B30RF70CF	55.45 ± 0.10 ^e^	9.51 ± 0.16 ^a^	25.31 ± 0.15 ^a^	5.83 ± 0.09 ^e^
B10RF90CF	54.04 ± 0.18 ^f^	9.77 ± 0.19 ^a^	25.14 ± 0.12 ^a^	5.53 ± 0.04 ^f^
BCF	53.42 ± 0.19 ^g^	9.90 ± 0.18 ^a^	25.06 ± 0.17 ^a^	5.39 ± 0.08 ^g^
B50RF50CF	56.74 ± 0.13 ^a^	9.25 ± 0.16 ^a^	25.49 ± 0.11 ^a^	6.13 ± 0.02 ^a^
B45RF50CF5CP	56.44 ± 0.25 ^a^	9.29 ± 0.22 ^a^	25.47 ± 0.15 ^a^	6.08 ± 0.09 ^a^
B45RF50CF5AP	56.49 ± 0.26 ^a^	9.27 ± 0.20 ^a^	25.46 ± 0.13 ^a^	6.09 ± 0.03 ^a^
B45RF50CF5BP	56.40± 0.18 ^a^	9.30 ± 0.22 ^a^	25.45 ± 0.19 ^a^	6.06 ± 0.04 ^a^

Biscuit formulations: Set I (BRF, B90RF10CF, B70RF30CF, B50RF50CF, B30RF70CF, B10RF90CF, BCF): biscuits from rice flour with 0%, 10%, 30%, 50%, 70%, 90%, and 100% chestnut flour. Set II (B50RF50CF, B45RF50CF5CP, B45RF50CF5AP, B45RF50CF5BP): biscuits from rice flour with 50% CF plus 5% fruit powders—chokeberry powder, açaí powder and blueberry powder. Data are presented as mean ± standard deviation of three independent analyses. Values with different letters within the same column are significantly different (one-way ANOVA, *p* < 0.05), while those with the same letter are not significantly different (*p* > 0.05).

**Table 5:** Macro- and microelements content of rice flour, chestnut flour, fruit powders and composite flours.

Sample	Macro and Microelement Content (mg/kg)							
	K	P	Mg	Ca	Fe	Zn	Mn	Cu
RF	1050.47 ± 0.10 ^g^	630.63 ± 0.37 ^g^	636.08 ± 0.11 ^g^	110.86 ± 0.12 ^g^	29.47 ± 0.09 ^g^	14.34 ± 0.09 ^g^	13.74 ± 0.08 ^g^	3.07 ± 0.08 ^g^
90RF10CF	1191.06 ± 0.16 ^f^	689.59 ± 0.28 ^f^	656.54 ± 0.09 ^f^	149.23 ± 0.09 ^f^	29.70 ± 0.04 ^f^	14.90 ± 0.03 ^f^	14.18 ± 0.28 ^f^	3.32 ± 0.02 ^f^
70RF30CF	1472.25 ± 0.12 ^e^	807.51 ± 0.32 ^e^	697.46 ± 0.04 ^e^	225.97 ± 0.18 ^e^	30.25 ± 0.02 ^e^	16.06± 0.07 ^e^	15.08 ± 0.16 ^e^	3.81 ± 0.09 ^e^
50RF50CF	1753.43 ± 0.18 ^d^	925.44 ± 0.26 ^d^	738.38 ± 0.18 ^d^	302.72 ± 0.19 ^d^	30.73 ± 0.12 ^d^	17.20 ± 0.13 ^d^	15.99 ± 0.09 ^d^	4.40 ± 0.13 ^d^
30RF70CF	2034.62 ± 0.16 ^c^	1043.36 ± 0.27 ^c^	779.29 ± 0.05 ^c^	379.46 ± 0.12 ^c^	31.22 ± 0.04 ^c^	18.30 ± 0.04 ^c^	16.88 ± 0.14 ^c^	4.93 ± 0.04 ^c^
10RF90CF	2315.80 ± 0.19 ^b^	1161.20 ± 0.28 ^b^	820.21 ± 0.19 ^b^	456.20 ± 0.14 ^b^	31.80 ± 0.10 ^b^	19.50 ± 0.16 ^b^	17.78 ± 0.08 ^b^	5.46 ± 0.02 ^b^
CF	2456.39 ± 0.15 ^a^	1220.25 ± 0.25 ^a^	840.67 ± 0.02 ^a^	494.58± 0.09 ^a^	32.07 ± 0.03 ^a^	20.06 ± 0.04 ^a^	18.18 ± 0.03 ^a^	5.72 ± 0.06 ^a^
CP	2514.21 ± 0.14 ^a^	2326.87 ± 0.26 ^a^	823.05 ± 0.25 ^b^	1256.66 ± 0.12 ^c^	84.57 ± 0.18 ^a^	135.05 ± 0.19 ^a^	7.48 ± 0.21 ^c^	9.46 ± 0.20 ^b^
AP	650.86 ± 0.19 ^c^	550.85 ± 0.22 ^b^	1560.82 ± 0.26 ^a^	2210.73 ± 0.25 ^a^	40.81 ± 0.09 ^c^	65.77 ± 0.26 ^b^	24.07 ± 0.09 ^b^	10.84 ± 0.26 ^a^
BP	994.78 ± 0.15 ^b^	82.55 ± 0.27 ^c^	675.07 ± 0.24 ^c^	1852.13 ± 0.22 ^b^	75.07 ± 0.19 ^b^	24.95 ± 0.23 ^c^	26.46 ± 0.25 ^a^	5.31 ± 0.13 ^c^
50RF50CF	1753.43 ± 0.18 ^b^	925.44 ± 0.16 ^b^	738.38 ± 0.08 ^d^	302.72 ± 0.19 ^d^	30.73 ± 0.12 ^d^	17.20 ± 0.13 ^d^	15.99 ± 0.09 ^b^	4.40 ± 0.13 ^b^
45RF50CF5CP	1826.62 ± 0.13 ^a^	1010.25 ± 0.25 ^a^	746.61 ± 0.03 ^b^	360.01 ± 0.14 ^c^	33.52 ± 0.09 ^a^	23.19 ± 0.04 ^a^	15.66 ± 0.20 ^c^	4.72 ± 0.05 ^a^
45RF50CF5AP	1732.56 ± 0.06 ^d^	921.45 ± 0.04 ^c^	784.61 ± 0.02 ^a^	407.71 ± 0.22 ^a^	31.30 ± 0.02 ^c^	19.74 ± 0.15 ^b^	16.48 ± 0.02 ^a^	4.78 ± 0.11 ^a^
45RF50CF5BP	1750.65 ± 0.08 ^c^	898.03 ± 0.28 ^d^	740.32 ± 0.09 ^c^	388.78 ± 0.14 ^b^	33.04 ± 0.06 ^b^	17.72 ± 0.07 ^c^	16.58 ± 0.09 ^a^	4.48 ± 0.17 ^b^

RF: rice flour; CF: chestnut flour; CP: chokeberry powder; AP: açaí powder; BP: blueberry powder. Set I (RF, 90RF10CF, 70RF30CF, 50RF50CF, 30RF70CF, 10RF90CF, and CF): rice flour with 0%, 10%, 30%, 50%, 70%, 90%, and 100% chestnut flour. Set II (50RF50CF, 45RF50CF5CP, 45RF50CF5AP, and 45RF50CF5BP): rice flour with 50% CF plus 5% fruit powders—chokeberry powder, açaí powder, and blueberry powder. Data are presented as mean ± standard deviation of three independent analyses. Values with different letters within the same column are significantly different (one-way ANOVA, *p* < 0.05), while those with the same letter are not significantly different (*p* > 0.05).

**Table 6:** Macro and microelement content of biscuits (control samples and fortified formulations).

Sample	Macro and Microelement Content (mg/kg)							
	K	P	Mg	Ca	Fe	Zn	Mn	Cu
BRF	889.26 ± 0.28 ^g^	389.26 ± 0.26 ^g^	489.22 ± 0.16 ^g^	79.14 ± 0.36 ^g^	18.45 ± 0.22 ^g^	10.32 ± 0.30 ^g^	8.42 ± 0.17 ^g^	1.23 ± 0.06 ^g^
B90RF10CF	1019.62 ± 0.21^f^	429.60 ± 0.03 ^f^	501.74 ± 0.08 ^f^	103.28 ± 0.12 ^f^	18.80 ± 0.05 ^f^	10.93 ± 0.05 ^f^	8.83 ± 0.03 ^f^	1.43 ± 0.02 ^f^
B70RF30CF	1280.21 ± 0.35 ^e^	510.32 ± 0.14 ^e^	526.76 ± 0.15 ^e^	151.24 ± 0.05 ^e^	19.51 ± 0.26 ^e^	12.17 ± 0.19 ^e^	9.66 ± 0.28 ^e^	1.83 ± 0.29 ^e^
B50RF50CF	1541.04 ± 0.29 ^d^	591.03 ± 0.06 ^d^	551.79 ± 0.07 ^d^	201.01 ± 0.14 ^d^	20.33 ± 0.09 ^d^	13.42 ± 0.14 ^d^	10.51 ± 0.14 ^d^	2.22 ± 0.07 ^d^
B30RF70CF	1801.66 ± 0.30 ^c^	671.73 ± 0.16 ^c^	576.80 ± 0.12 ^c^	249.69 ± 0.09 ^c^	21.09 ± 0.12 ^c^	14.66 ± 0.20 ^c^	11.33 ± 0.08 ^c^	2.62 ± 0.17 ^c^
B10RF90CF	2062.38 ± 0.27 ^b^	752.41 ± 0.11 ^b^	600.88 ± 0.06 ^b^	297.54 ± 0.15 ^b^	21.75 ± 0.09 ^b^	15.97 ± 0.12 ^b^	12.20 ± 0.11 ^b^	3.01 ± 0.04 ^b^
BCF	2192.83 ± 0.29 ^a^	792.84 ± 0.07 ^a^	614.24 ± 0.21 ^a^	322.92 ± 0.26 ^a^	22.22 ± 0.12 ^a^	16.59 ± 0.04 ^a^	12.64 ± 0.09 ^a^	3.21 ± 0.05 ^a^
B50RF50CF	1541.04 ± 0.09 ^c^	591.03 ± 0.06 ^c^	551.79 ± 0.07 ^d^	201.01 ± 0.14 ^d^	20.33 ± 0.09 ^c^	13.42 ± 0.14 ^d^	10.51 ± 0.11 ^c^	2.22 ± 0.04 ^d^
B45RF50CF5CP	1621.71 ± 0.16 ^a^	686.93 ± 0.14 ^a^	567.50 ± 0.19 ^b^	258.42 ± 0.05 ^c^	23.24 ± 0.16 ^a^	19.56 ± 0.07 ^a^	10.42 ± 0.06 ^c^	2.58 ± 0.03 ^b^
B45RF50CF5AP	1528.13 ± 0.07 ^d^	598.12 ± 0.09 ^b^	604.60 ± 0.28 ^a^	306.45 ± 0.02 ^a^	20.96 ± 0.24 ^b^	16.23 ± 0.23 ^b^	11.24 ± 0.02 ^b^	2.69 ± 0.06 ^a^
B45RF50CF5BP	1546.27 ± 0.15 ^b^	574.10 ± 0.02 ^d^	560.73 ± 0.14 ^c^	288.47 ± 0.17 ^b^	23.08 ± 0.01 ^a^	13.95 ± 0.05 ^c^	11.41 ± 0.12 ^a^	2.39 ± 0.12 ^c^

Biscuit formulations: Set I (BRF, B90RF10CF, B70RF30CF, B50RF50CF, B30RF70CF, B10RF90CF, BCF): biscuits from rice flour with 0%, 10%, 30%, 50%, 70%, 90%, and 100% chestnut flour. Set II (B50RF50CF, B45RF50CF5CP, B45RF50CF5AP, B45RF50CF5BP): biscuits from rice flour with 50% CF plus 5% fruit powders—chokeberry powder, açaí powder and blueberry powder. Data are presented as mean ± standard deviation of three independent analyses. Values with different letters within the same column are significantly different (one-way ANOVA, *p* < 0.05), while those with the same letter are not significantly different (*p* > 0.05).

**Table 7:** Total phenolic content, total flavonoid content and antioxidant activity measured by FRAP of rice flour, chestnut flour, fruit powders and composite flours.

Sample	TPC (mg GAE/100 g d.s.)	TFC (mg QE/100 g d.s.)	FRAP (μM Fe^2+^/g d.s.)
RF	59.10 ± 0.14 ^g^	44.46 ± 0.19 ^g^	8.09 ± 0.22 ^g^
90RF10CF	67.54 ± 0.29 ^f^	51.69 ± 0.27 ^f^	8.73 ± 0.17 ^f^
70RF30CF	86.23 ± 0.08 ^e^	68.42 ± 0.24 ^e^	10.42 ± 0.30 ^e^
50RF50CF	104.72 ± 0.12 ^d^	83.34 ± 0.22 ^d^	12.08 ± 0.12 ^d^
30RF70CF	124.52 ± 0.25 ^c^	100.24 ± 0.18 ^c^	13.50 ± 0.27 ^c^
10RF90CF	143.16 ± 0.19 ^b^	115.76 ± 0.08 ^b^	14.92 ± 0.09 ^b^
CF	152.44 ± 0.08 ^a^	123.57 ± 0.27 ^a^	16.18 ± 0.14 ^a^
CP	5512.44 ± 0.24 ^a^	3966.48 ± 0.36 ^a^	449.63 ± 0.27 ^a^
AP	5060.04 ± 0.25 ^c^	2442.03 ± 0.31 ^c^	377.90 ± 0.15 ^c^
BP	5482.71 ± 0.33 ^b^	3711.44 ± 0.14 ^b^	412.08 ± 0.19 ^b^
50RF50CF	104.72 ± 0.12 ^d^	83.34 ± 0.22 ^d^	12.08 ± 0.12 ^d^
45RF50CF5CP	377.41 ± 0.15 ^a^	280.40 ± 0.14 ^a^	32.51 ± 0.25 ^a^
45RF50CF5AP	354.62 ± 0.35 ^c^	203.86 ± 0.28 ^c^	28.64 ± 0.06 ^c^
45RF50CF5BP	376.04 ± 0.28 ^b^	266.48 ± 0.30 ^b^	30.46 ± 0.04 ^b^

RF: rice flour; CF: chestnut flour; CP: chokeberry powder; AP: açaí powder; BP: blueberry powder. Set I (RF, 90RF10CF, 70RF30CF, 50RF50CF, 30RF70CF, 10RF90CF, and CF): rice flour with 0%, 10%, 30%, 50%, 70%, 90%, and 100% chestnut flour. Set II (50RF50CF, 45RF50CF5CP, 45RF50CF5AP, and 45RF50CF5BP): rice flour with 50% CF plus 5% fruit powders—chokeberry powder, açaí powder, and blueberry powder. Data are presented as mean ± standard deviation of three independent analyses. Values with different letters within the same column are significantly different (one-way ANOVA, *p* < 0.05), while those with the same letter are not significantly different (*p* > 0.05).

**Table 8:** DPPH inhibition (%) of rice flour, chestnut flour, fruit powders and composite flours.

Sample	Dilution of Extract	DPPH Inhibition (%)
RF	1:100	28.14 ± 0.17 ^g^
90RF10CF	1:100	31.64 ± 0.23 ^f^
70RF30CF	1:100	37.87 ± 0.25 ^e^
50RF50CF	1:100	44.86 ± 0.13 ^d^
30RF70CF	1:100	52.25 ± 0.24 ^c^
10RF90CF	1:100	59.94 ± 0.08 ^b^
CF	1:100	62.62 ± 0.12 ^a^
CP	1:2000	143.65 ± 0.26 ^a^
AP	1:2000	126.63 ± 0.07 ^c^
BP	1:2000	131.72 ± 0.18 ^b^
50RF50CF	1:100	44.86 ± 0.13 ^d^
45RF50CF5CP	1:100	51.36 ± 0.26 ^a^
45RF50CF5AP	1:100	50.27 ± 0.09 ^c^
45RF50CF5BP	1:100	51.02 ± 0.03 ^b^

RF: rice flour; CF: chestnut flour; CP: chokeberry powder; AP: açaí powder; BP: blueberry powder. Set I (RF, 90RF10CF, 70RF30CF, 50RF50CF, 30RF70CF, 10RF90CF, and CF): rice flour with 0%, 10%, 30%, 50%, 70%, 90%, and 100% chestnut flour. Set II (50RF50CF, 45RF50CF5CP, 45RF50CF5AP, and 45RF50CF5BP): rice flour with 50% CF plus 5% fruit powders—chokeberry powder, açaí powder, and blueberry powder. Data are presented as mean ± standard deviation of three independent analyses. Values with different letters within the same column are significantly different (one-way ANOVA, *p* < 0.05), while those with the same letter are not significantly different (*p* > 0.05).

**Table 9:** DPPH inhibition (%) of biscuits (control samples and fortified formulations).

Sample	Dilution of Extract	DPPH Inhibition (%)
BRF	1:100	27.54 ± 0.09 ^g^
B90RF10CF	1:100	30.29 ± 0.13 ^f^
B70RF30CF	1:100	37.49 ± 0.21 ^e^
B50RF50CF	1:100	44.89 ± 0.28 ^d^
B30RF70CF	1:100	51.25 ± 0.06 ^c^
B10RF90CF	1:100	59.29 ± 0.31 ^b^
BCF	1:100	62.45 ± 0.23 ^a^
B50RF50CF	1:100	44.89 ± 0.28 ^d^
B45RF50CF5CP	1:100	50.36 ± 0.32 ^a^
B45RF50CF5AP	1:100	48.57 ± 0.05 ^c^
B45RF50CF5BP	1:100	49.14 ± 0.16 ^b^

Biscuit formulations: Set I (BRF, B90RF10CF, B70RF30CF, B50RF50CF, B30RF70CF, B10RF90CF, BCF): biscuits from rice flour with 0%, 10%, 30%, 50%, 70%, 90%, and 100% chestnut flour. Set II (B50RF50CF, B45RF50CF5CP, B45RF50CF5AP, B45RF50CF5BP): biscuits from rice flour with 50% CF plus 5% fruit powders—chokeberry powder, açaí powder and blueberry powder. Data are presented as mean ± standard deviation from three independent analyses. Values with different letters within the same column are significantly different (one-way ANOVA, *p* < 0.05), while values with the same letter are not significantly different (*p* > 0.05).

**Table 10:** SAXS parameters of flours, fruit powders and biscuit formulations.

Sample	Median of the Distribution, nm	Surface Equivalent Mean Size, nm	Goodness of Fit
RF	850.25	40.31	1.06
CF	912.98	134.13	1.10
CP	821.78	35.68	1.00
AP	784.48	25.69	1.11
BP	757.67	24.31	1.18
BRF	866.67	71.49	1.04
B50RF50CF	884.21	87.44	1.03
BCF	864.74	55.36	1.10
B45RF50CF5CP	847.38	52.85	1.00
B45RF50CF5AP	867.71	65.59	1.00
B45RF50CF5BP	872.78	68.91	1.05

RF: rice flour; CF: chestnut flour; CP: chokeberry powder; AP: açaí powder; BP: blueberry powder; BRF, B50RF50CF, BCF: biscuits from rice flour with 0%, 50% and 100% chestnut flour; B45RF50CF5CP, B45RF50CF5AP, B45RF50CF5BP: biscuits from rice flour with 50% CF plus 5% fruit powders—chokeberry powder, açaí powder and blueberry powder.

## Data Availability

Original data and contributions presented in this study are provided in the article or Supplementary Materials and are accessible at the University of Life Sciences “King Mihai I” from Timisoara.

## Supplementary Materials

The following supporting information can be downloaded at https://www.mdpi.com/article/10.3390/foods14234074/s1: Table S1: Comparison of scattering angles peaks (2θ) from Wide-Angle X-Ray Scattering of flours and fruit powders and Table S2: Comparison of scattering angles peaks (2θ) from Wide-Angle X-Ray Scattering of biscuit formulations.
