# Supplementary material for: Current Trends in Gluten-Free Biscuit Formulation Using Rice Flour Enriched with Chestnut Flour and Fruit Powders
Source: Foods. 2025 Nov 27;14(23):4074. doi: 10.3390/foods14234074 (PMC12692167; doi:10.3390/foods14234074)
Supplement: Supplementary file 1 [file foods-14-04074-s001.zip › foods-3986312-supplementary.pdf]

**Table S1.** Comparison of scattering angles peaks (2θ) from Wide Angle X-Ray Scattering of flours and fruit powders

| Scattering angle, 2θ | | | | |
|---|---|---|---|---|
| RF | CF | CP | AP | BP |
| 4.8 | 4.8 | 5.4 | - | - |
| 14.8 | 11.3; 14.8 | - | - | - |
| 16.7; 22.2 | 16.7; 18.1; 21.7 | 19.9 | 18.9 | 17.3; 19.8 |
| - | 23.7; 29.7; 30.6 | - | - | - |
| 32.3 | 32.3; 37.1; 38.7 | 33.5 | 33.5 | 33.4 |

RF: rice flour; CF: chestnut flour; CP: chokeberry powder; AP: açaí powder; BP: blueberry powder

**Table S2.** Comparison of scattering angles peaks (2θ) from Wide Angle X-Ray Scattering of biscuit formulations

| Scattering angle, 2θ | | | | | |
|---|---|---|---|---|---|
| BRF | BCF | B50RF50CF | B45RF50CF5CP | B45RF50CF5AP | B45RF50CF5BP |
| 3.5; 5.7 | - | 3.6 | 3.6 | 3.9 | - |
| 14.2 | 14 | 14.1 | 13.8; 14.1 | 13.9 | 14.0 |
| | 16.9 | 16.6 | - | - | 17 |
| 19 | 19.1 | 19.1 | 19.0; 19.1 | 19.1 | 19,1 |
| 23.5 | 23.6 | 23.6 | 23.3;23.5 | 23.5; 25.5 | 23.5 |
| 27.0 | 27.0 | 27.0 | 26.4; 27.0 | 27.1 | 26.9 |
| 28.5 | 28.4 | 28.4 | 28.5 | 28.5 | 28.4 |
| 30.1 | 30.1 | 30.1 | 29.8; 30.2 | 30.2 | 30.0 |
| 31.6 | 31.6 | 31.6 | 31.1; 31.4; 31.6 | 31.6 | 31.6 |
| 33.1 | 33.1 | 33.1 | 32.7; 33.1 | 33.1 | 32.9 |
| 36.2 | 36.2 | 36.2 | 36.2 | 36.3 | 36.1 |
| 37.2 | 37.3 | 37.2 | 37.3 | 37.2 | 37.0 |
| 38.7 | 38.7 | 38.5 | 38.4 | 38.7 | 38.5 |
| 39.7 | 39.7 | 39.7 | 39.4; 39.7 | 39.7 | 39.7 |
| 41.0 | 41.0; 41.3 | 40.9 | 40.8; 41.1 | 41.1 | 41.0 |
| 43.4 | 43.5 | 43.4 | 43.2 | 43.5 | 43.3 |
| 45.9 | 45.9 | 45.8 | 45.7 | 45.9 | 45.8 |
| 48.0 | 48.2 | 48.0 | 47.8 | 48.0 | 47.9 |
| 50.3 | 50.3 | 50.3 | 50.1 | 50.3 | 50.1 |
| 50.9 | 51 | 50.9 | 50.9 | 51 | 50.9 |
| 52.3 | 52.4 | 52.3 | 51.9; 52.4 | 52.4 | 52.3 |

BRF, B50RF50CF, BCF: biscuits from rice flour with 0%, 50% and 100% chestnut flour; B45RF50CF5CP, B45RF50CF5AP, B45RF50CF5BP: biscuits from rice flour with 50% CF plus 5% fruit powders – chokeberry powder, açaí powder and blueberry powder.
